# A Philosophical Treatise on Practical Medicine

**Published:** 1840-07

**Authors:** 


### Art. IV.

*Traité Philosophique de Médecine Pratique.* Par A. N. Gendrin, d.m., Médecin de l'Hôpital de la Pitié. Tomes I-II.—*Paris*, 1838-9. 8vo, pp. 703, 694.

*A Philosophical Treatise on Practical Medicine.* By A. N. Gendrin, m.d., Physician of La Pitié. Vols. I-II.—*Paris*, 1838-9.

M. Gendrin has held a distinguished place among modern pathologists since the publication of his valuable researches into the anatomical history of inflammations. A second great work from his pen, following the former at a distance of thirteen years, and bearing the ambitious title of *A Philosophical Treatise on Practical Medicine*, awakens therefore no common expectation. The qualities that most conduce to the advancement of all inductive science are patience and exactness in the observation of facts, and skill in so arranging and analyzing them as to discover their true relations and import. These qualities, M. Gendrin has shown that he possesses; and he enjoys ample scope for their exercise in the wards of La Pitié. We naturally anticipate, from his maturer labours, the verification or correction of such general principles and precepts as have been previously current,—if not the development of new and still more comprehensive truths.

The first two volumes only of this treatise have yet appeared. Exclusive of a short introduction, they are entirely occupied with the subjects of Hemorrhage and the Diacrises, or diseases depending on alterations of secretion. As in the introductory chapter, however, the author unfolds the method according to which he proposes to class and investigate all the various forms of disease that fall within his plan, we shall bestow a cursory notice upon this part of the work before we proceed to the important practical topics, to which it is our intention to restrict the present article.

Like most writers who assume a scientific tone, M. Gendrin begins at the beginning, and tries his hand at formal definitions. This is both a proper and a necessary procedure, when we have to do with terms and phrases which might otherwise be obscure, or vague, or liable to different interpretations. But we hold it to be a useless expense of ingenuity when applied to familiar things which all men apprehend and identify alike.

His first paragraph professes to explain what is meant by the state of health. Now this is more easily understood than described—more easily described than defined. We do not think the sentence worth extracting. A part of the difficulty of framing a short formula, which shall express all the conditions implied in that term, and nothing more, arises from the fluctuation which those conditions may undergo without transgressing the limits of sound health. The author truly observes that "the state of health is not an absolute state; it is not the same at every instant of time; it is not identical in all individuals; it is not even incompatible with certain permanent defects in the organs of the body: there are congenital or acquired infirmities, which impede or prevent the action of important organs, but do not destroy the equilibrium of functions by which life is continued, and health maintained." (p. 1.)

Nor is his definition of disease more instructive or satisfactory. We do not require or profit by any definition either of health or of disease in the abstract. These terms are each the converse of the other, and alike intractable to scholastic handling. If we could fix in a few words the essential idea of a state of health, it would be enough afterwards to say that disease consists in any deviation from that state. But our conception of disease is perplexed rather than helped, when we are told that "a malady is the union of unwonted phenomena occurring simultaneously or in succession, and in a certain relation to each other, which phenomena are developed in consequence of an alteration in the physiological condition of organs." (p. 3.)

The following remark which precedes this definition, but has a close relation to its subject, is pregnant with profound meaning; and, although the truth which it contains is usually learned at last by those who see much of disease and who reflect on what they see—we fear its full import is too often unperceived by the student, and neglected by the careless and the routine practitioner: "Diseases derive their *origin* from the direct action of injurious causes; but their *subsequent* course, phenomena, and results are determined by those vital laws which regulate also the healthy functions of the whole system." (p. 2.)

M. Gendrin makes Bichat's division of the functions into those of organic life and those of animal life, subservient to his classification of diseases. The general comparison which he draws between the forms of disease that pertain respectively to these two primary divisions of the functions, is diluted by so many words, and so much repetition, as to tire without satisfying the attention. Its substance may be compressed into a short compass.

The diseases that belong to organic life bear these peculiar characters: They have a definite seat, and they alter, for a time or permanently, the structure of the parts they occupy. The functions of relation suffer in these disorders in a secondary manner only, and in consequence of injury done to the organs that are their instruments. Maladies of this class may cause death by disturbing or preventing the vital functions, through changes thus wrought in their principal organs. In the investigation of these maladies, the physician is not restricted to those symptoms which the patient himself happens to be conscious of, or to exhibit externally, but he explores by his own senses the physical condition of the organs upon the continued functions of which depends the continuance of life.

The diseases that belong to animal life, on the other hand, are not necessarily connected with change of structure; and do not, primarily, modify the function of nutrition. They present alterations in the functions of the nervous and muscular systems alone. When they prove mortal, they are so indirectly, by destroying the relations of the individual to those objects which are indispensable to his existence, or by exhausting the vital organs through the continuance of morbid action. It is seldom that, in studying this class of disorders, the physician can gather information from a physical examination of the bodily organs. He is under the necessity of forming his judgment according to the symptoms declared to him by the patient, and the aberrations of function that manifest themselves to his observation.

If all the acts of organic and animal life were reciprocally independent of each other, the division of the functions which they accomplish in the animal economy would be easy and exact. But these acts have organs in common, and a mutual reaction. The parts that minister to the functions of animal life are built up and sustained by the functions of organic life. Nevertheless, an important and a well-marked distinction may be observed between most of the numerous maladies that occur without compromising, primitively, the functions of relation and the maladies, also many in number, that implicate these latter functions only.

M. Gendrin separates, then, all the diseases of which he proposes to treat into two great divisions. The first of these divisions includes the maladies that consist in alteration of the functions of organic life. The second comprehends the maladies that consist essentially in alteration of the functions of the life of relation. These two grand divisions comprise together nine classes of maladies, formed according to the nature of the morbid states, as estimated by their constant phenomena; whether these phenomena reveal themselves to clinical observation, or are discovered by a skilful examination of the bodily organs and of the results of functional actions during life and after death.

"Class i. Hemorrhages. The diseases of this class are placed in the first rank, inasmuch as they consist in alterations in the organs and functions which are concerned in the circulation of the blood, which conduce therefore the most directly to the maintenance of life, and which are the first among all the organs and all the functions in living beings of all kinds. Hemorrhages are also disorders, the phenomena of which are the most easily apprehended, and deviate the least from the conditions of health. It is besides important to consider them in the outset, for these diseases are in themselves causes, symptoms, constitutive phenomena, epiphenomena, and even modes of termination and of treatment of several other diseases.

"Class ii. Alterations of Secretion, or Diacrises. These diseases are morbid actions of greater complexity than hemorrhages: they are often also causes, essential or accidental symptoms, epiphenomena, and modes of termination and of treatment of many other diseases. Less simple than the hemorrhages in their constituent conditions, they nevertheless resemble them in being dependent on the functions of the circulating system, which conveys to the secreting organs the elements of the elaboration performed by them; but they possess this condition in particular, that they are the direct result of a morbid change in the process of elaboration itself.

"Class iii. Phlegmasiæ. These maladies, by reason of the greater number of their elements, are less simple than those of the preceding classes. They have this in common with them, that they are always connected with modifications of the circulation, differing in intensity only from those that constitute the hemorrhages and the diacrises. Often they are the cause or the effect either of hemorrhages or of diacrises: frequently also they are modified during their course by some one of those forms of disease supervening spontaneously or purposely produced.

"Class iv. Fevers or Pyrexiæ. This class comprehends diseases characterized by disturbance of the functions of the circulating system, and frequently of the nervous system; to which are added lesions belonging to the three preceding classes, with this peculiarity, that these lesions, and the functional derangements of the circulating and nervous systems occur together or in succession according to an order for which we do not perceive in themselves a sufficient reason.

"Class v. Modifications in the Nutrition of Organs, or Anomalotrophies. This class comprises all the diseases in which organs or parts of organs are so modified in respect to their nutrition as to approximate in their structure to the texture of other parts—or as to assume an unusual bulk—or as to diminish in bulk and to lose the natural consistence of their tissues—or as to lose their relations of contiguity or of continuity with other parts. These af fections are often consecutive to many of the diseases of the preceding classes; they are also themselves the cause, more or less immediate, of many of those diseases—with which they are frequently thus complicated.

"CLASS VI. HETEROSARCOSES, OR THE FORMATIONS OF ACCIDENTAL TISSUES. The production, in the midst of our organs, of accidental tissues, which are nourished, increase, and are destroyed in a manner altogether specific, forms the character of these diseases. Most commonly they are independent in their production upon any antecedent malady belonging to the clases already described: but very frequently, by the mere circumstance of their development, they occasion certain of those maladies, which influence in their turn the progress of the primitive disease.

"CLASS VII. CACHEXIÆ. The seat of these maladies is in all the organs, for they effect, primitively, the general organs common to all the tissues. Their symptoms sometimes are expressive of languor and general atony in the action of all the organs, marked especially by loss of colour, diminished consistence, dryness and diminution of heat in all parts of the body. Sometimes they only bespeak a perversion of the general function of nutrition, giving rise to similar lesions in many organs that differ essentially in structure and vitality. The cachexiæ may produce, as symptoms, the greater part of the diseases of the preceding classes; and these diseases are often in their turn the main causes of cachexia.

"CLASS VIII. NEUROSES. These diseases consist in idiopathic modifications of the functions of the whole nervous system—or of parts of that system—considered as the agent of sensation, volition, and nervous excitement or innervation. The form and the importance of these maladies vary according to the functions that are deranged, and according to the extent and nature of those functional derangements. The character proper to these maladies, as well as to those of the succeeding class, is the impossibility of penetrating beyond the functional lesion, so as to refer it to a change in the texture of organs.

"CLASS IX. VESANIÆ. These constitute the last class of diseases—and consist essentially in alterations of the intellectual functions, independent on any discoverable idiopathic lesion of the brain or its appendages.

"The first seven of these classes belong to the first division of diseases, which embraces lesions of the functions, the actions, or the organs of the life of nutrition. The second division includes the eighth and ninth classes.

"There are some maladies which it is impossible to bring strictly within either the one or the other of these divisions. It does not, however, appear necessary to frame an intermediate division for the reception of these mixed diseases. They are all neuroses, and exhibit, in combination with phenomena belonging to derangements in the functions of organic life, marks of a still greater disturbance in the functions of animal life. There is a practical utility in uniting them with the other neuroses—inasmuch as they present similar therapeutic indications, and require similar methods and means of treatment." (pp. 17, et seq.)

We pass no judgment, at present, upon the foregoing system of classification. The great difficulty will occur in parcelling out the multiform disorders of the human frame into species, and in assigning to each species its due place in its proper class. What degree of skill M. Gendrin may bring to an attempt in which all nosologists that have gone before him have been foiled will appear as his work proceeds. We scarcely venture to hope, however, that he will be much more fortunate in this respect than Cullen, who closes his elaborate endeavour to form a complete methodical arrangement of diseases, with a "Catologus morborum a nobis omissorum, quos omisisse fortassis non oportebat." The science of medicine has made a vast stride in advance since his day; but there are yet many parts of it too obscure and unsettled to admit of a perfect and systematic classification.

M. Gendrin divides the First Part of his work, comprehending his First Class of Diseases, HEMORRHAGES, into four books. The following are the subjects treated under each:—

BOOK I. Of Hemorrhages in general.

BOOK II. Of Hemorrhages which take place from exhalant surfaces, or through the channels of secretion.

A. *Hemorrhages of mucous membranes.*
1. Epistaxis.
2. Hemoptysis or bronchial hemorrhage.
3. Gastro-hemorrhage.
4. Entero-hemorrhage.
5. Hematuria.
6. Uretro-hemorrhage.

B. *Cutaneous Hemorrhages.*
1. Hematidrosis, or bloody sweat.

C. *Hemorrhages of serous membranes.*

BOOK III. Hemorrhages into the substance of Tissues, or Interstitial Hemorrhages.
1. Hemorrhoids.
2. Apoplexy, or hemorrhage within the skull or vertebral canal.
3. Pneumo-hemorrhage.

BOOK IV. Morbid modifications of natural or functional hemorrhage.
1. Menstruation.
2. Dysmenorrhœa.
3. Metro-hemorrhage.
4. Utero-placentar hemorrhage.

On the present occasion, it is our purpose to confine our attention to the subjects treated of in the first and last books; reserving for another opportunity the notice of the other diseases included in this most important class of maladies.

I. OF HEMORRHAGE IN GENERAL. The escape of the blood from the vessels that contain and carry it, whereby it is poured out of the system or deposited in the cavities of the body or in the interstices of its tissues, has been taken by most writers on the subject as the single essential circumstance in that morbid state which is designated by the name of hemorrhage. Certain phenomena have indeed been noted as frequently preceding the extravasation of blood, and certain other phenomena as frequently following it. M. Gendrin assuredly takes the true and philosophic view of the matter, when he includes, as parts of the entire malady, those unnatural conditions of the system which prepare for the extravasation, those which accompany it, those which succeed it, and those which repair its effects.

There are four elementary morbid conditions, by the succession or combination of some of which, all hemorrhages are marked—viz. 1, Polyæmia, or a state of general plethora; 2, Hyperæmia, or local congestion of blood; 3, Extravasation of blood, or hemorrhage strictly so called; 4, Olygæmia, or, according to the usual but less exact nomenclature, anæmia.

These conditions are familiar to the experienced physician. The author's elaborate description of each contains nothing new or peculiar.

The first two of these are often precursors of the malady; the last is never connected with it but as its consequence.

Hemorrhages are always composed of at least two of these elements—local hyperæmia, and extravasation of blood. Often a third morbid element concurs, namely, general plethora or polyæmia. And the loss of blood may throw the patient into a state of olygæmia. This consecutive morbid state imprints in its turn peculiar characters on the fresh hemorrhage which may take place during its existence, and thus it becomes another element of the malady. It takes the place of the sanguine plethora, with which it is incompatible: and the hemorrhage is still made up of three elements—local congestion, extravasation of blood, and olygæmia. This constitutes what has been called passive hemorrhage; and it is often symptomatic of other morbid conditions.

There is scarcely any part of the body which may not be the *seat* of hemorrhage; but those organs are most liable to it of which the texture is the most delicate and the least compact, which are provided with the greatest number of red blood-vessels, and of which the vascular network is the least deeply involved in the proper tissue of the organ. Hence hemorrhage from the mucous membranes is the most common of all; and especially from those which possess in the highest degree the anatomical peculiarities just noted. Next to the mucous membranes, in respect of liability to hemorrhage, comes the skin. Seldom does idiopathic hemorrhage proceed from the serous surfaces. It takes place, however, from the follicles or canals that belong to secreting organs; and M. Gendrin inclines to the opinion (which many familiar facts might be adduced to corroborate) that the blood in all these cases escapes through the same channels as give exit to the several secretions. Hemorrhage into the substance of the tissues is also of frequent occurrence; and these tissues are the most obnoxious to this kind of lesion which contain the greatest number of red vessels.

The author is very full—not to say verbose and redundant—upon the *etiology* of hemorrhage. All the influences that produce, or tend to produce, either general plethora or local congestion are obviously to be considered as predisposing causes of hemorrhage. The *ingesta*, the *circumfusa*, the *applicata*, the *acta*, the *excreta*, the *animi pathemata*, form so many heads under which causes of this kind are discussed.

Among the *ingesta*, a full and stimulant diet and the continued use of exciting liquors may justly be reckoned as direct causes of general plethora. That tea or coffee, when taken habitually, have (as the author supposes) any such effect is in our opinion a mere piece of theory which all experience serves rather to confute than to confirm. Neither are we aware of any facts warranting the belief that preparations of iron ever tend to occasion hemorrhage by producing sanguine plethora. It may be admitted that such ingesta as exercise a specific stimulating action upon certain organs will be likely to produce congestion of blood in those organs, and therefore a disposition to hemorrhage. But probably there are not many substances which can be proved to possess such a special power. M. Gendrin mentions emmenagogue drinks and drastic purgatives, as respectively calculated to determine hemorrhage from the uterus and from the rectum.

The *circumfusa* include chiefly certain qualities of the atmosphere. Dry and elevated situations and a continued high temperature, whether natural or artificial, conduce to sanguine plethora, and consequently to hemorrhage. A proclivity to hemorrhage is noticeable among the inhabitants of hot climates: the menstrual flux is more abundant. We can readily appreciate the tendency to hemorrhage produced in certain persons by great and sudden meteorological changes which cause a sensible variation in the weight of the atmosphere and in its electric condition, and which thus exercise an intelligible influence upon the circulation. The author quotes a remarkable illustration of this, recorded by Dr. Mead.

"In the year 1687, Dr. Pitcairn being at a country-seat near Edinburgh, on a fairer day than usual at that season, and the sun looking reddish, was seized at nine in the morning, the very hour of the new moon, with a sudden bleeding at the nose, after an uncommon faintness. And the next day, on his return to town, he found that the barometer was lower at that very hour than either he or his friend Dr. Gregory, who kept the journal of the weather, had ever observed it: and that another friend of his, Mr. Cockburn, Professor of Philosophy, had died suddenly at the same hour by an eruption of blood from the lungs; and also five or six others of his patients were seized with different hemorrhages."

We have long surmised that the occurence, at certain periods, of apoplexies in such numbers as almost to give an epidemic character to that disease—a fact of which no hospital physician can be ignorant—may often be attributed to the immediate influence of atmospheric changes upon the circulation of individuals disposed to cerebral hemorrhage by previous disease of the blood-vessels. The connexion between ascertainable meteorological phenomena and the prevalence of particular disorders opens a wide and interesting and almost untrodden field of research, to which we earnestly invite attention.

The effect of a very cold atmosphere as a determining cause of hemorrhage is well known; and the common explanation of this is probably the true one, namely, that the cold operates by repelling the blood from the superficial capillaries, and accumulating it in the deeper-seated vessels. It was observed that, in the Russian campaign in 1812, many of the French soldiers were attacked with bleeding from the nose, mouth, and various parts of the body.

The *applicata* consist of mechanical violence, chemical irritants, hot and relaxing applications, such as poultices, compression by means of ligatures or otherwise.

The *acta* include all those movements of the body which imply long-continued or strong effort. These are points which require no particular notice.

Under the head of *excreta* the author remarks that every act of secretion is preceded by hyperæmia of the secerning part; and that when the process is forced into undue activity, blood is apt to make its appearance in lieu of or mixed with the proper product of secretion. Another way in which the excreta may be connected with hemorrhage is when some natural or long-continued discharge of blood, or of materials furnished by the blood, is suppressed, while the whole mass of blood continues to be replenished as fast as before the drain ceased, and plethora is the necessary consequence.

*Moral emotions* also—such as alarm, anger, anxiety—have often, however the fact may be accounted for, been succeeded by hemorrhage. After noticing these, M. Gendrin goes on to consider the influence of *sex*, *age*, *temperament*, *and the accomplishment of certain functions*, in the production of hemorrhage.

It is notorious that women are more subject to these disorders than men. This partly resolves itself into their greater liability to hemorrhage *by deviation* resulting from interruption to the periodical escape of the blood or its materials in menstruation. M. Gendrin affirms, however, that, independently of this function, females are more prone to those conditions of local or general plethora which constitute a predisposition to hemorrhage: so that this proclivity is evident not only during the period of life between puberty and the final cessation of the catamenia, but also before and after that period. He believes those authors to have been mistaken who have considered males to be more frequently afflicted, especially in advanced life, to hemorrhois and hematuria. It would require a large tabular record of facts to determine this question; which, after all, is not of much obvious or practical importance. We question the accuracy of the latter part of the following remark :

"The greater aptitude of hemorrhage manifested by females during almost the whole of their lives, is a predisposing circumstance which renders them also more subject to plethora and hyperæmia. The same aptitude enables them to bear bloodletting better than men, and teaches us the propriety of insisting on that therapeutic measure with less reserve in the disorders of women." (p. 59.)

With respect to *age*, hemorrhages take place at every period of life; but it has long been matter of observation that they happen more at certain ages than at others, and that they proceed from certain parts of the body more commonly at one time of life than at another. The period of puberty, the epoch at which the growth of the body is just completed, the time at which its maturity may be said to terminate, are the three periods of life when hemorrhage is most common. Again, in newly-born children the digestive organs are most apt to be the seat of hemorrhage; in children and adolescence, the nasal cavities; in adult age the lungs and intestines; in the middle portion of life, the colon and rectum; then the brain; and in old age, the tendency to cerebral hemorrhage is combined with a disposition to the discharge of blood by the urinary organs.

M. Gendrin maintains that it is not exact to say, with preceding authors, that the sanguine *temperament* is the most favorable to hemorrhage, but rather the sanguineo-lymphatic, which is characterized by a considerable development of the organs of circulation, with a certain degree of fulness of person, and of laxity of the cellular tissue and of the integuments. This temperament is more frequently met with in females, and it is always connected with a high degree of nervous susceptibility, which favours the operation of other occasional causes of congestion and hemorrhage.

The *functions*, upon the failure or irregularity of which may depend the production of hemorrhage, are those of menstruation and parturition; and the manner in which general plethora or local congestion may become the consequence of a defective or an excessive elimination of blood from the system in connexion with these functions, requires no further exposition.

Sleep, according to M. Gendrin, is an undoubted promoter of hemorrhage. In the majority of cases of hemorrhage, in persons predisposed to that form of disease, the bleeding comes on during sleep. This is a fact of which we were not cognizant. We even presume to question the correctness of the statement.

That the disposition to hemorrhage descends often from parent to child is unquestionable. We conceive that this disposition may be transmitted just as those peculiarities of shape, feature, and complexion are transmitted that constitute family likenesses. The same plan or pattern of conformation, the same qualities of texture and tissue, occurring in the offspring, confer a predisposition to the reproduction in them of the disorders of the parent. But we are not aware of any facts which go to show that the hemorrhagic tendency is hereditary, in that wider and more remarkable sense in which scrofula, gout, or insanity, are truly said to be hereditary,—the proclivity to these complaints descending from father to son, or overleaping a generation and reappearing in the grandson, although there may be no discoverable resemblance in the cast of countenance, the colour of the hair, eyes, and skin, or the general configuration of the bodily frame. It is, however, an important fact that a disposition to hemorrhage on the application of slight causes is apt to run in families; and that sometimes it is so strong as to impress a very singular and unfortunate character, constituting what has been called the hemorrhagic diathesis, upon the individuals who derive their origin from a common progenitor.*

In considering the pathological conditions which act as predisposing or occasional causes of hemorrhage, the author again brings forward the subject of plethora; and this habit of repeatedly exhibiting the same thoughts and circumstances under different heads, imparts to some portions of his work a degree of confusion and perplexity which is much to be regretted. His general facts are for the most part well selected, and his inferences from them are usually sound and just, but they are not skilfully or clearly disposed. And as there can be no pretence to novelty in the doctrines we have hitherto been examining, we look upon this loose and diffuse mode of discussing them as a very serious defect, seeing that what is naturally expected from a writer who assumes the office of setting a great subject in order, is, that he should present that subject under its most plain and perspicuous aspect. In perusing the present work, we cannot help feeling that M. Gendrin is occasionally wearisome. Several pages still further onwards we reach a paragraph, of which the subject matter is "en quoi consiste la plethore."

Hemorrhages are among those disorders which generate, the more frequently they happen, a disposition to their own recurrence. When the system has been accustomed to losses of blood, and to the renovation of the lost fluid, it is thereby rendered more than commonly susceptible of

* See a marked instance of this in No. xvii. of this Journal, p. 247.

local or general fulness, and therefore of hemorrhage, which redresses such fulness. Hence we find that artificial bloodletting, frequently repeated, tends to produce plethora and consecutive hemorrhage.

Whatever morbid condition implies great disturbance of the circulation of blood, or a mechanical impediment to its course, will be apt to have hemorrhage as one of its symptoms or accidental effects. And when the blood is poured forth from a ruptured vessel, it becomes a symptom of the accident or malady which led to the rupture.

We pass by the author's observations on the *proximate causes* and the *nature* of hemorrhages, and the chapters devoted to their *diagnosis* and *prognosis*, as containing only the common-places of these topics, involved, as usual, in a superfluity of words; and we come, in the last place, to the *general therapeutics* of hemorrhage.

When hemorrhage is symptomatic of some disease which impedes the free passage of the blood, or effects a breach of its containing vessels, or modifies the quantity of the blood and the contractility and texture of the vessels and tissues, the treatment applicable to the hemorrhage will commonly merge in that which is required by the antecedent disease.

In idiopathic hemorrhage, the first point the physician has to consider is, whether the bleeding should be left to itself, or whether he should attempt to staunch it? The answer to these questions must be collected from an investigation of the component elements of the malady, and of the organs which it affects. The principal morbid element of the disease consists in hyperæmia or plethora, and these conditions often find their cure in the extravasation of the blood, which is harmless and salutary whenever it does not exceed the limits required by the degree of the preceding congestion, and implies no damage to the organs in which it occurs. In such cases, an *expectant* treatment is the only treatment that is rational. This does not mean that no measures whatever are to be taken, but that our endeavours are to be limited to preventing the discharge of blood from becoming excessive or irregular, to warding off all causes that are likely to increase it, to keeping the patient cool and tranquil, and to correcting any obvious disorder in the digestive organs, or in the circulating or nervous systems. To attempt the direct suppression of the bleedings while the plethora that occasioned it remained unredressed, would be to incur the hazard of producing some other and less innocent form of hemorrhage.

The expectant treatment becomes unsuitable, and even dangerous, when important organs are implicated or threatened; or when, either from the age of the patient, or from his previous state of health, the mere loss of blood is likely to be injurious. We have then to consider the treatment proper to be employed for removing the state of general plethora. Artificial abstraction of blood, and artificial congestion, are the first among the agents curative of that state.

Bloodletting, which operates by diminishing the quantity of blood in the vessels, and by rendering it less stimulant, because less rich in fibrine and colouring matter, is still to be restrained within certain limits. When carried to excess, or repeated too often, it generates the very condition it is intended to correct. Nor is bloodletting alone sufficient for the object in view. Its influence must be assisted by repose of body and tranquillity of mind, by a temperate and anstimulant diet, and by the less direct depletion of the blood-vessels effected by remedies that augment the natural secretions.

The author here discovers that groundless horror of purgative medicines which has long formed, and which we believe continues, though in a mitigated degree, still to form, so singular a feature in the French school. Purgatives, he argues, are stimulants; and, in a state of general plethora, we must employ them with much reserve, lest we produce congestion, hemorrhage, inflammation, in the parts on which they act. He prefers, therefore, active diuretics, of which he affirms, 1st, that they are more effectual (a proposition wherein we place no credence); and, 2dly, that they are not attended (why, he neglects to explain,) with similar risks. He specifies two drugs as being eminently serviceable, digitalis and nitre. From one to three grains daily of the former, in substance, prove diuretic, he tells us, in a high degree, and may be administered for a long time together, without diminution of their influence upon the kidneys, and without inconvenience. The action of the nitrate of potash as a diuretic is more speedy, but perhaps less sustained; it must be given to the amount of three or four drachms, or even more, every day, and in that quantity its only unpleasant effect is its disagreeable taste.

The author next takes into consideration the means proper for relieving *local congestion.* Where hyperæmia exists on the exterior of the body, the obvious cure would seem to lie in emptying the gorged vessels of the part, by the direct removal of blood. But, besides the state of general plethora which may be present and constantly renew the local congestion in spite of bleeding, there may be some special cause in operation that keeps the vessels full. We must guard against allowing the mode of taking away blood to encourage and maintain congestion, by the local irritation it produces.

The general rules in respect to treatment may be thus laid down: We must first counteract and annul the condition of general plethora when it exists. This being effected, the local congestion will often subside spontaneously, either at once or by degrees. But when no indication of general plethora is perceptible, and the local hyperæmia persists, we combat the latter. And the question immediately occurs, whether we are to do this by emptying, in as direct a way as we can, the turgid vessels of the affected part, or by means of distant revulsion or derivation.

M. Gendrin replies to this question in substance as follows: If the hyperæmia has lasted for some time, and manifests no disposition to increase, we may as well operate at once upon the seat of the congestion. But here we should avoid, as much as possible, the use of means likely to increase or keep up the congestion. For the same reason we should make the loss of blood considerable. The irritation caused by leeches, or the suction of cupping-glasses, will be apt to sustain the local fulness, unless the quantity of blood removed by them is sufficient to countervail and overbalance that tendency. We have a practical illustration of the propriety of this rule in the effect of a few leeches applied frequently to the groins or vulva, in promoting deficient menstruation, compared with the effect of a large number applied at one time in relieving uterine congestion. If, on the other hand, the hyperæmia appears to be augmenting, or shows a tendency after some diminution to return, then it will be better to solicit congestion to some other part. When the morbid congestion has resulted from deviation or suppression of some previous local flux, we must endeavour to restore the original discharge, especially if this be more harmless than the consecutive hyperæmia.

The measures employed for the purpose of effecting such a diversion of the congestion are general bleeding, which indirectly withdraws the blood from the suffering organ, and the distant application of what are called rubefacients, sinapisms, hot water, cupping-glasses. The new hyperæmia caused by the latter expedients takes place at the expense of the morbid hyperæmia, and conduces to its relief. Care must be taken to establish the artificial congestion at a sufficient distance, to renew it frequently, or to maintain it until the morbid congestion has disappeared.

Another mode of accomplishing the same object—traceable to an early period, but seldom used, we apprehend, at present—is that of applying ligatures upon the limbs or compression to certain arteries of the body. Ligatures necessarily impede the return of the venous blood from the extremities towards the heart. They must not be so tight as materially to hinder the course of the blood through the arteries. The blood is thus shut up in the veins and capillary vessels of the limb. Hence the quantity contained in the vessels of the rest of the body is diminished, and feebleness of the circulation with faintness is rapidly induced. By these means the vessels of the diseased part are indirectly emptied; and a similar effect is produced directly by compression of the arteries that supply it with blood. We can seldom avail ourselves in practice of this expedient; yet in some instances, where life is ebbing under rapid hemorrhage, it is both practicable and of the utmost advantage.

When general plethora or local congestion has been effectually remedied, extravasation of blood seldom continues. The cure of most hemorrhages consists in the removal of one or both of these elements of the whole morbid condition. Therapeutic measures are, however, sometimes employed with the view of staunching the flow of blood. They are supposed to have the property of constringing or obstructing the channels of the hemorrhage or of diminishing the activity of the circulation in the diseased organ. So long as there is much topical congestion remaining, such measures are dangerous; for they tend to increase the irritation and other mischievous consequences of the hyperæmia, which a continuance of the hemorrhage would relieve. This hazard belongs especially to mechanical or chemical styptics made use of to restrain external bleeding.

When outward applications of this kind are inappropriate or impossible, internal medicines are given with the same object of arresting the escape of the blood. They are believed to operate through some sympathetic relation between the bleeding part and the alimentary tube, or by effecting some change in the blood itself. M. Gendrin regards this as a mere hypothesis; and affirms that we have no evidence of the efficacy of such medicines, exhibited internally, in restraining hemorrhages. Such scepticism, however, is groundless, and the result of mere inexperience. It is probable that his theoretical persuasion of the inutility of drugs in such cases, or his inability to perceive in what manner they can influence the disposition to hemorrhage, has prevented him from making the requisite trial of those which are most in repute. No English physician can be ignorant of the styptic qualities of the acetate of lead. Its modus operandi is more questionable. There are some other forms of medicine which we may reasonably suppose to act by their astringent property upon the capillary vessels or the tissues by which these are surrounded. Of this kind are the numerous vegetable substances that contain gallic acid, which passes undecomposed through the blood, and reappears in the excretions, and especially in the urine. That certain forms of uterine hemorrhage are controllable by the ergot of rye, administered internally, is also beyond dispute.

The author's observations respecting the influence of cold and of the state of syncope in staying hemorrhages, call for no particular comment.

When oligæmia is the consequence of repeated or excessive losses of blood, it becomes in its turn an object of remedial treatment; and M. Gendrin's recommendation of steel and bitter medicines, of moderate exercise in a pure and dry air, and of friction to the surface of the body, is such as all practical men in this country will appreciate and approve. Not so, we think, the *nimia cura* which, over and above these measures, would direct specific remedies against the several symptoms that arise out of the oligæmia—digitalis, for example, to quiet the palpitations that are apt to attend it; alkaline potions for the dyspepsia; diuretics for the œdema of the ankles. These are mere accidents proceeding from the deficiency of red blood, and will disappear when that want is repaired. Indeed, they are more likely, in our judgment, to be aggravated than relieved by the means which our author has proposed.

Upon the whole we confess that the First Book of M. Gendrin's work has somewhat disappointed our expectations. That the doctrines advanced in it are most of them sound, and supported by reason as well as by experience, we willingly admit. And we do not complain of them because they are not new; novelty not being the desideratum in such undertakings, but truth. We are instinctively put upon our guard against a writer whose main boast it is that he has discoveries to announce which had eluded the sagacity of all his duller predecessors. Such vain and dishonest pretenders are not unknown to the medical literature of our neighbours across the channel; but M. Gendrin is of another order. What we most want at present is that the vast mass of facts relating to disease, accumulated through a long series of bygone ages, and prodigiously augmented during the last quarter of a century, should be turned into the propositions and precepts of true science; that the mute language of established phenomena should be rightly interpreted and clearly expressed, and rendered profitable for our daily use. We do not say that M. Gendrin is not qualified to supply this want; but we are bound to declare that in expounding those general views of hemorrhage which have formed the subject of our present criticism, we have found him diffuse even to dulness, and strikingly deficient in that lucid arrangement and gracefulness of style which are requisite, in order to display even solid knowledge to advantage, and without which it is difficult to arrest the attention or command the interest of the majority of readers.

II. Of Menstruation and its Disorders; and of Uterine Hemorrhage. It is not our intention to notice all the author's opinions on each of the subjects treated of in the Fourth Book of M. Gendrin's work, since in many cases these in no way differ from those received as current in the profession; but we shall confine ourselves to the discussion of those points which are new, or in which M. Gendrin differs from the received doctrines.

THEORY OF MENSTRUATION. Under the first category of novelties we find the attempt to trace the menstrual function to certain conditions of the ovaria. The means of demonstration adopted are elaborate, and the doctrine itself deserves attention. The only rational means, says M. Gendrin, of coming at the proximate cause of menstruation is to ascertain the exact state of the internal organs of generation, with relation to the appearance and periodical return of the catamenia, and to compare these results with the different circumstances known to occur in the establishment, the suspension, or the final cessation of menstruation. With this purpose the author details the particulars of five dissections made in women dying during the menstrual period, and then examines the state of the ovaria, 1, prior to puberty; 2, after the menstrual flux has ceased at the critical age; 3, the state of the ovaria of women who menstruate regularly; 4, their state in such as have interrupted menstruation; 5, their state in those in whom suppression has been consequent on chronic maladies. As instances of death during the catamenial flow are comparatively rare, and the appearances rarely described, we shall give an abridgment of the whole of the five cases detailed by M. Gendrin.

"CASE I. A woman, æt. thirty, subject to mental excitement at the menstrual period, hung herself, as the husband stated, at the epoch of menstruation. The mucous membrane of the vagina and the cervix uteri were very vascular. The substance of the uterus was much injected; its cavity contained mucus tinged with blood. The inner membrane was lined by fungiform villi of a grayish colour, especially at the fundus, which villi were a line in length and best seen under water. The *right* Fallopian tube and ovary were natural. In the latter were three Graafian vesicles, from half a line to two lines under the surface. The left Fallopian tube was dilated, so as to be a line in diameter, and was filled with a bloody mucus, which extended to the funnel of the fimbriæ which it filled. The left ovary was injected at its surface, and about three lines in the middle of which a jagged edged rupture was visible about *a line in diameter.* Its cavity could contain a hemp-seed; its walls were intensely red. It was obviously a broken Graafian vesicle. Four Graafian vesicles existed at various depths in the same ovary.

"CASE II. A girl, three of whose relations had committed suicide, flung herself out of a window while menstruating, fractured her skull, and died in an hour. The hymen was entire. A slight quantity of blood was found in the vagina. The mucous membrane of the vagina and the os tincæ were deeply injected. The uterine cavity contained blood and mucus. Its surface was covered by villi, which, when viewed by the microscope, were red and fungiform; their extremities bulbous, one line in length: no vessels were seen on them. The Fallopian tubes were filled with red mucus, the left for half its length, the right in its whole extent, up to the fimbriæ, which were fixed to the ovary. This organ was covered by a thick mesh of admirably-injected vessels. A small circular jagged orifice led to a cavity two lines in diameter, the walls and bottom of which were of a vivid red colour. The network of vessels in the ovarian surface increased in density until it became uniform in tint near the rupture. Three vesicles, two of which equalled hemp-seed, the third a pin's head, in size, were in this ovary. The largest was near the surface, the smallest about one line and a half beneath it. The left ovary contained five vesicles of the size of a millet-seed, at varying depths: there was a yellow spot in which no cicatrix could be detected. The red mucus in the Fallopian tube was examined with a lens magnifying 150 times, but no organized body could be discovered in it.

"CASE III. A girl, æt. twenty-seven, at the third day of the menstrual period, had her arm torn off by machinery. The catamenia, which usually lasted six days, were suppressed, and she died in thirty-six hours after the accident. There was no particular injection of the mucous membrane of the vagina or cervix uteri. The uterine cavity contained a small quantity of sanguinolent mucus. Its inner surface had but a feeble show of villi at the fundus. The Fallopian tube contained a little mucus, of a pale rose colour. The right ovary had two imperfectly-healed cicatrices, one of which was nippled and led to a small central cavity. The other was of a yellowish tint. A small red cavity, one line and a half deep, was found under this scar. A few vessels crept over the ovary; there were five unbroken Graafian vesicles in the left, and only one in the right ovary.

"Case iv. A girl, twenty years of age, had for three days laboured under double pleurisy and pericarditis. On the fifth day of her malady the catamenia appeared, but became suppressed in twenty-four hours, and she died three days after. The vagina and cervix uteri were natural; a clot of half-coagulated blood was found in the uterus. No villosities were discoverable by a lens on the uterine walls. The two Fallopian tubes were filled throughout their extent with colourless mucus. The right ovary had a small rupture, one line and a half in diameter, leading to a cavity of the diameter of two lines, the walls of which were red and flocculent, and its orifice surrounded by a vascular meshwork. A single Graafian vesicle was found in this ovary. The left ovary had no cicatricula nor Graafian follicle: it was only half the natural size, and weighed but twenty-three grains.

"Case v. A woman, æt. forty-four, who had borne three children, was attacked with apoplexy, leaving incomplete palsy of the left arm and constant headach. She was regular, and her menses appeared on the 11th July: on the 12th she was attacked by giddiness, loss of sensibility, &c., and died in the night. There was effusion of blood in the lateral ventricles of the right side. A coagulum filled the uterus, which was half as large again as natural. The inner surface was filled with villosities apparently vascular. The left Fallopian tube was dilated and filled with red mucus. Its fimbriæ were fixed to the ovary, on the surface of which was a little rupture two lines in diameter. Bloody mucus penetrated through this and filled the cavity. The cavity examined under water equalled one line in diameter, and was the centre of a vascular spot, which was four to five lines in diameter. In the right ovary there was no lesion; it contained two vesicles in the ordinary state. The left had three of the size of hemp-seed." (Tom. ii. pp. 18-21.)

Here, then, we have five cases of death during menstruation, in each of which there is a rupture of a sac, presumed to be a Graafian vesicle. The inner membrane of the uterus has a villous surface developed on it. The Fallopian tube is found dilated and filled by a sanguinolent mucosity. All this apparatus is periodically called forth and is periodically extinguished; and if we look to the length of that period it will be found to increase our wonder, since the rapidity is no less remarkable than the extent of the changes produced. A woman in good health menstruates seven days, and then remains free twenty-one, so that this healing of a cicatrix, and the clearing away of the villi lining the uterus, together with the preparation and obtrusion of a fresh vesicle for the forthcoming period, are to be carried on all in the twenty-one days of inter-menstruation. This intense and incessant activity, this mushroom growth and decay, is of itself sufficiently startling, and would make us examine with great caution before we adopt the theory of M. Gendrin.

We proceed with the examination of the ovaria in their immature or impubic state; in the modification produced in them by the cessation of menstruation at the critical period; and by the suppression caused by disease.

There is no canal in the Fallopian tube at ten years of age. M. Gendrin admits, however, the great difficulty of ascertaining the presence of a canal in the Fallopian tube in very young subjects, owing to the tenuity and shortness of this organ. He asserts that no Graafian vesicle was found in the ovaria previous to puberty, except in three instances of girls rather more than twelve years old, who at the same time had never menstruated. In each of these the Fallopian tubes were perforated, and had the relations seen at a riper age with the ovaria. The vesicles, of the size of a pin's head, were deeply imbedded in an undeveloped ovary. There were from one to four in each.

In women who have outlived the critical age, as it is termed, the ovaries are known to become atrophied. M. Gendrin says that they are converted into a condensed cellular tissue, containing many cavities, while the outer layer, or tunica albuginea, assumes a fibrous texture. No traces of the cicatrices of corpora lutea are visible on its shrivelled and irregular surface. The Fallopian tubes also become thin, the canal large, as its fimbrial end, and impermeable at its uterine extremity. These latter assertions are founded on injections of quicksilver made on two subjects only, and they are further qualified by the difficulty M. Gendrin felt in determining how far the permeability of the Fallopian tubes was owing to the laxness of texture which permitted the stylet of the syringe to force a new path or to find the old one.

M. Gendrin has assured us that for years he has neglected no occasion in examining, at his hospital, the state of the organ we are now discussing. His generalizations want neither vigour nor extent; they may be said in common parlance to be sweeping: it is to be lamented, therefore, that this portion of his treatise should rest on two facts on which the author himself cannot rely.

With regard to the state of the ovaria in those who have regular menstrual periods, he says that the Graafian vesicles are nearer the ovarian surface in proportion to their size; that cicatrices are found varying from the freshest red in colour, with a most decided irregular cavity, to a pale yellow spot, scarcely disturbing the level or polish of the ovarian surface. "In every instance where the patient has menstruated from one to more weeks before death, an occurrence which in acute maladies is anything but rare, we have always found the cicatrix more vascular and recent as the period was shorter." (p. 25.)

The ovaria in women who have interrupted menstruation either contain no vesicles or only small ones deeply imbedded in them. In two instances only, small cells with red smooth sides, filled with a sanious matter, existed; and around their orifices there was the same kind of vascular patch as in the rest. The one of these patients died of encephalitis, after a three months' suspension of the catamenia, the other of typhus, after two months' cessation of the periodical discharges.

M. Gendrin asks whether in these instances the same inflammatory action which is apparent in the mature and superficial vesicle can also exist in the unripe and deep-seated one? "Or, perhaps, the disposition of parts is the result of a modification similar to that which accompanies the menstrual flux, with this difference, however, that this action is profound and, as it were, latent." (p. 26.)

The influence of chronic diseases may be such that the flux shall occur at distant intervals or be quite regular, or it may cease entirely. Each of these three states M. Gendrin asserts may be recognized from the appearances in the dead body. When it has long ceased, the Graafian vesicles are either non-existent or few, small, and deep-seated; the ovaria themselves being small, irregular, unvascular, and atrophied. This wasting is further recognized by the extreme diminution of the ovarian vessels, and coincides always with the absence of the Graafian vesicle.

When the catamenia appear at irregular intervals there are generally Graafian vesicles in the ovaria. In one instance, in which the flux occurred only six days before death, there was a reddish yellow cicatrix on the surface of the right ovary. The two ovaria are never atrophied in the same degree; most often one is sound and contains vesicles, while the other is decayed and without them.

As for those in whom chronic disease does not interfere with the regularity of the catamenia, the ovaria exhibit the same lesions as occur in acute maladies.

Such is the scaffolding of facts raised, by means of which M. Gendrin means to erect his philosophic structure, or, to drop metaphor, to arrive at the following conclusions—showing the special conditions of the ovaria under which the "menstrual hemorrhage can alone occur:"

"A. Conditions necessary for the capacity to menstruate:

"1. The normal development of the ovaria and Fallopian tube, which takes place at puberty and lasts till the critical age.

"2. The presence of Graafian vesicles in the ovaria, which are more mature the nearer they are to the surface.

"B. Conditions necessary for the appearance and actual presence of the flux:

"1. The presence at the surface of the ovary of one or two inflamed cells, evidently resulting from a rupture of a vesicle which is in the progress of cure by the reparative inflammation.

"2. The dilatation of the Fallopian tube, and its position still very near the ovary.

"3. The repletion of the Fallopian tube with mucus more or less bloody.

"4. The presence of sanguinolent mucus, or grumous blood in the uterus.

"5. The manifestation of fungiform villi, which are probably vascular, on the surface of the uterus.

"6. The turgescence of the vascular system of the ovary, Fallopian tube, the uterus, and vagina, in the last of which organs it may be recognized during life; also the turgescence of the mammæ.

"C. Conditions where the menstrual flux has terminated, and during the intervals between the periods:

"1. The greater or less cicatrization of the ovarian alveoli.

"2. Corpora lutea, the remains of a cicatrization completed, which De Graaf had attributed to a previous fecundation.

"3. The progressive development of Graafian vesicles, and their progressive approach to the ovarian surface.

"D. Conditions of the menstrual flux, as yet unestablished before puberty, or having ceased after the critical age:

"1. The absence of the ovarian vesicle.

"2. The non-development, or the atrophy of the ovary.

"E. Conditions for interrupted or regular menstruation:

"1. The Graafian vesicles small in number, and removed from the ovarian surface.

"2. When the flux has taken place, the existence of ovarian alveoli more o less cicatrized, and the absence of Graafian vesicles of a certain degree of development, situated near the ovarian surface.

"F. Conditions of menstruation suppressed for a certain time by some morbid cause:

"1. The total absence of Graafian vesicles, whether deeply or superficially seated.

"2. A greater or less degree of atrophy of the ovaria and Fallopian tubes.

"G. Conditions where menstruation has never been established:

"1. Absence of Graafian vesicles, and the atrophy of the ovaria and Fallopian tubes." (pp. 27-9.)

The general conclusions to be drawn from these researches are: that menstruation is only a periodical phenomenon of a function commencing with puberty and ending with the critical age—which function consists in the production and development of vesicles in the ovary, that is, of a matured ovum, which is periodically brought forward, either to be expelled with the menstrual flux by the uterus, or to be destroyed by rupture and inflammation. This last act being the termination of the formation and of the evolution of each vesicle, and of the contained ovule, cannot be continuous: it is accomplished at regular epochs; and it is to this act, to which the hemorrhagic turgescence of the genital organs is attached, of which menstruation is the result. (p. 29.)

The disposition of the Fallopian tubes, which was found in Case II. fixed to the ovary during menstruation, suddenly interrupted by death. The known function of this organ is to receive the ovule. The active consociation of uterus and ovary, marked in the act of menstruation in this case precisely as in conception, by uterine turgescence and "travail organique;" the constant presence of inflammation and a broken vesicle in the ovary—all these circumstances lead to the presumption that, on this occasion, the Fallopian tube receives the ovule and transmits it to the uterus.

It is not true, and Home had asserted it already, that the rupture of a Graafian vesicle is dependent on fecundation alone; this occurs at each menstrual period. (p. 33.)

The menstrual flux is connected, therefore, with the maturation ana periodical expulsion of an ovum. But the proximate cause of the discharge is genital hyperæmia and the formation of uterine villi, which Jörg first described. The analogy between spermatic impregnation and these phenomena varies as to what becomes of the ovule: if it is destroyed in the ovarian cell, that is a special termination of menstruation and not of conception; if it passes into the uterus, not having the conditions of life in it, it is not fixed but passed off with the flux. (p. 33.)

The theory which has been just adduced, if the facts be complete, must modify profoundly, as M. Gendrin himself asserts, our notions of the function of menstruation. It accounts, in some measure, for the acknowledged fact that impregnation generally takes place most readily just after the menstrual period. It connects, as cause and effect, the uterine periodical flux with the ovarian function, and so throws some light on the connexion between menstruation and the capacity of fecundation.

The chief objection which occurs to us as militating against M. Gendrin's views arises from the following consideration: If at every menstruation an ovule escapes from a ruptured Graafian vesicle, the cicatrices of corpora lutea must be much more numerous than we find them to be. Supposing a woman to menstruate once in every lunar month, thirteen ovarian ruptures will occur annually; and unless we suppose that the wound so caused at each period is completely healed before a new solution of continuity takes place, we should find the ovaria of a menstruating female, one would think, indented and filled with numerous excavations in different states of reparation. That such is not the state of the ovaria, as seen by M. Gendrin, his cases which we have abstracted prove; that such ought to be their state, if the observations of the supposed discoverers in this branch of physiology are to be relied on, might be easily shown. These observations tend to prove that the ovarian wound caused by the escape of an ovule is either recognizable in its open state or as closed by a substance called corpus luteum, *for nearly two years.* If this be true, we ought, in adopting M. Gendrin's hypothesis, to find in this period nearly twenty-six scars or wounds, many of which would be open, while others would exhibit the various states of a cavity undergoing the process of reparation peculiar to a ruptured Graafian vesicle. We have ourselves repeatedly seen the corpus luteum in those who have died after childbirth, and can corroborate the fact, attested by Meckel and many others, that it is distinctly visible at least for twelve months. Indeed, reference to all the original facts bearing on this point, an excellent digest of which may be seen in Burdach's Physiology, prove that the healing of a ruptured Graafian vesicle is a long affair, and that the cicatrices are visible for so extended a period of time, that were ruptures of these sacs as frequent as menstrual periods, the ovaria must be in a state of perpetual open wound—" a sort of internal issue."

Another objection, though in our opinion by no means a conclusive one, may be urged from the alleged fact of the number of ova being limited to about thirty in the human female: although this opinion of Haller's has obtained the sanction of Burdach, it cannot, we think, be proved. It has arisen from indirect inference rather than from direct evidence. Few women have borne more than thirty times, including miscarriages: this fact, together with another, namely, that in proportion as the human female approaches to the critical age, so the visible number of Graafian vesicles diminishes, has probably led to the above surmise of Meckel and others. In the researches of Dr. Martin Barry, noticed in our Seventeenth Number, there are some most elaborate and apparently accurate microscopical observations, from which it would seem that the ovaria of all the mammalia are filled with minute ovisacs, as he terms them, as thick as are the ova in the roe of a fish; and which, he asserts, are constantly developing and decaying. The objection resulting from the supposed limitation in the number of ova in the ovary may be looked on as inconclusive. We incline to the belief that this number is variable, without pretending to affix to it the narrow limit of Haller and Leveret; nor the enlarged precision of Roederer, who estimated it at fifty; nor the countless multitude assigned by Dr. Martin Barry.

If there is such a succession of ova lost, and wounds inflicted, as M. Gendrin supposes, we may ask, What becomes of the assertions of

Haller, Ruysch, De Graaf, Santorini, Duvernay, Heister, and Meckel, that they especially directed their attention to the discovery of corpora lutea and never found them but where fecundation had taken place? "I have examined," says Meckel,* "the ovaria of pregnant women, as well as of the female of various classes of animals, in more than 200 cases, and have found, without a single exception, that the number of young and that of the corpora lutea exactly corresponded." This assertion of Meckel's excludes the formation of corpora lutea from any other sources than fecundation. Indeed, he combats the contrary notion by stating that "yellow substances may be found in the ovary, but every yellow substance is not, therefore, a corpus luteum."

Blumenbach, Cuvier, and Home have given instances, however, of the coexistence of corpora lutea with the presence of an intact hymen; and, besides the examples adduced by M. Gendrin, others are readily found in the medical collections where these yellow bodies have been seen in the ovaria of children.

We have thus stated some of the objections which arise against the sweeping generalizations of M. Gendrin. We give these credit for ingenuity and for their foundation on some valuable facts; but we say that though the profession are indebted to him for calling their attention to the subject of the menstrual function, yet his case is not proven. It is very probable that the theory of Cullen, that the ovary is the governing organ in the menstrual impetus, will prove ultimately the true one; but a complete review of the subject, so as to unite the positive observations of Haller, Meckel, &c. with those of modern embryologists, is still in reserve for science.

That M. Gendrin should have found the ovaria ruptured in five cases where menstruation was present is a remarkable coincidence. In a communication read at the Medico-Chirurgical Society by Dr. Robert Lee, last year, a similar occurrence was stated to have been met with in two instances. We want but a few more such facts to establish M. Gendrin's hypothesis; or at least to give it the aspect of a law rather than that of a mere coincidence; and until these are obtained we would again repeat "not proven."

And now for a few words on M. Gendrin's originality. In Dr. Churchill's Outlines of the Principal Diseases of Females (p. 63), it is noticed that Dr. Friend, in his Emmenologia, alludes, so early as 1729, to the influence of the ovaries in menstruation. Dr. Power, in his Essays on the Female Economy, attributes menstruation entirely to the action of the ovaries. It has long been known that on removal of the ovaria the menstrual flux ceases. Such a case is related by Pott, and another more recently by Dr. Montgomery. In our Sixteenth Number (p. 531) we have quoted precisely this very theory of M. Gendrin's, as entertained by Mr. Jones, who has also referred to dissections made by himself and friends in proof of its correctness. Mr. Jones does not inform us whether these observations were made without any knowledge of M. Gendrin's opinions. We find, too, that Mr. C. Negrier, the Professor of Midwifery at Angers, has claimed this discovery of M. Gendrin.† It appears that

* Handbuch der Menschlichen Anatomie. Vol. iv. p. 686.

† Gazette Médicale de Paris. No. xlii, 1839, p. 672.

he wrote a Memoir on this very subject, and read the same at the Medical Society of Angers so early as 1832. This memoir was transmitted to Paris in April, 1838, and read by Adelon, Paul Dubois, Ollivier, and others; it was also left at the publishers many months for the purpose of *being copied*—all this before 1839, the date of M. Gendrin's second volume. Nevertheless, that gentleman denies that he ever saw M. Negrier's work; asserts that he does not know whether the two sets of observations are parallel; and that, if M. Negrier made his discovery in 1832, he, M. Gendrin, made his in 1828.*

Having thus laid down his physiological principles, M. Gendrin enters on the subject of functional disorders of menstruation, at first generally, throwing into the front of his more special descriptions remarks on topics bearing on most of the maladies of the genital system.

As menstruation is a function originating and ending, says M. Gendrin, in the ovary (p. 38), the hemorrhage attending it is to be looked on as an intermitting phenomenon, differing in nothing from any other habitual sanguineous flux; and curative indications founded on this alone are, therefore, insufficient. This statement, it will be seen, though a direct consequence of his theory, is in fact only the repetition of the commonly received notion, that menstruation is not a simple discharge from the uterus, but the effect of a foregone constitutional act, the seat of which is unknown. All the phenomena of menstruation but the mere exit of the secretion may and do often take place periodically.

Amenorrhœa. This disease, according to our author, is the result of suspended ovarian function, that is, we presume, there is no disengagement of an ovum from its Graafian vesicle. In scanty menstruation the ovule is disengaged, but the fault is in the uterus. (p. 39.) These observations savour too much, we think, of system. The cause of the deficiency in the ovarian function is traced to agencies acting beyond the sphere of the ovary: 1, to the influence of the nervous system; 2, of the circulating system; 3, to alterations in the blood itself:—facts which we admit in every point save in the doubtful inference that these changes first affect the ovary, and through that the menstrual function.

It is well known that a moral shock will suspend the catamenia—for what will not the nervous system affect? No less true is it that diseases of the heart and vascular excitements of various kinds disturb the periodical discharge; and most pathologists are agreed that the quality of the blood is altered in amenorrhœa, while some view this alteration as the prime cause of the malady. The curative indications, therefore, in amenorrhœa, are to look to the cause of the disease, and not to found a treatment on means the object of which shall be merely to force an uterine discharge. Derivative bleedings, strong emmenagogues, are, says M. Gendrin, hurtful; and we agree with the general statement, affixing,

* A celebrated bibliopolist, now dead, used to designate the monomania under which certain rich and respectable elderly gentlemen laboured, of abstracting from their friends' shelves and cabinets, the editio princeps, or a proof above price, as "condiddling," a term which, as combining a refined classical affix with the strenuous vernacular of the hundreds of Drury, marks the distinction of "abstraction" by those who have received a liberal education and those who have not. M. Raspail has, we observe, very strong misgivings as to the means by which the scientific ardour of the "confrèrerie" is nourished, as he has compelled his publisher to append, by way of index, to the last edition of his valuable work, the date at which every sheet of MSS. was sent to him!

however, this limit to it, that in any functions composed of many steps the induction of one will draw on the rest. Thus, simple irritation of the os uteri will often as effectually produce labour-pains in a pregnant womb as rupture of the membranes; and so likewise will warm hip-baths by simply determining to the hypogastrium, give rise first to a scanty sanguineous discharge and then to the regular development of menstruation. Nevertheless, M. Gendrin's rule is both scientific and practical, and infinitely safer and better based than the silly ingenuity evinced by Amussat in his recommendation of an exhausted cupping-glass to the os uteri!

Imperfect Menstruation. In this state the uterus and not the ovary, says our author, is in fault; for there are all the signs of the ovarian nisus in the weight and tension in the pelvis, and the scalding and heat in the act of evacuating the bowels and bladder. This being satisfactorily determined, M. Gendrin would have us bleed generally or locally, and apply emollients and vapour baths, to solicit the rigid fibre of the plethoric; while in the weak and cachectic the bloodletting might with advantage be omitted. Here is just one of the points in which a specious theory may induce pernicious consequences. Whether the person suffering from scanty menstruation be weak or robust, the cause, according to M. Gendrin, is still a defect in the vascular system of the uterus, and the remedies are, consistently with this view, confined to such as will unload the vessels.

Now we confidently appeal to general experience, whether, in the scanty flux of debility, the surest and the most rapid recoveries do not take place by means of a course of chalybeates—of medicines, therefore, which, were M. Gendrin's practical inferences true, ought to increase the uterine phlogosis.

M. Gendrin dwells at some length on the effect of acute and chronic diseases on menstruation. Generally speaking, when an acute malady suspends the flux, we have all the signs of plethoric oppression, and the blood is determined to that organ which is the seat of the intercurrent malady. Here M. Gendrin recommends the sound practice of attending to the new disease, and not troubling ourselves as to "deriving" to the uterus. When the superinduced disease does not suspend but merely diminishes the discharge, then we have the old theory—the crambe decies repetita—that the uterus and not the ovary is in fault, and you are, therefore, to attend both to the uterus and to the intercurrent disorder. We would advise the practitioner, whether the malady suspends entirely or merely diminishes the menstrual flux, to treat the malady which has thus interfered with the function, and regard everything else as quite secondary to that.

With respect to chronic maladies, M. Gendrin has but a few words; but they indicate a great truth little appreciated in this country, where active practice is synonymous with judicious practice. "In chronic diseases the cases are few which require the use of active measures, and we may always without risk postpone our remedies; the rather because the therapeutics of chronic diseases, to be efficacious, should be slow, prolonged, and chronic, like the malady itself." (Vol. ii. p. 47.) "Acute disease, acute treatment—chronic disease, chronic treatment," may recall to many the ponderous witticism of Dr. Johnson, "who drives fat oxen must himself be fat," still we maintain that there are few truths in practical medicine more important than that here enunciated by M. Gendrin as to the chronic treatment, as he happily terms it, of chronic diseases.

We will not enter into the discussion as to whether the presence of the menstrual flux should interfere with the use of powerful remedial measures, such as bleeding, emetics, and purgatives; nor will we stop to analyze the chapters on Dysmenorrhœa, under which term the author has included vicarious as well as painful menstruation; on which heads M. Gendrin is prolix, without adding anything to our knowledge: we pass on, therefore, to the consideration of uterine hemorrhage, a subject occupying more than half of M. Gendrin's second volume.

Metro-Hemorrhage. Under the term of metro-hemorrhage M. Gendrin includes: 1, Excessive menstruation; 2, Hemorrhage occurring under conditions of the frame which usually precludes it, as, for example, hemorrhage before puberty, or after the critical age, or during gestation and lactation; 3, Puerperal hemorrhage. Of these divisions the last, or puerperal hemorrhage, claims, from its interest and importance, the greatest attention. We hasten, therefore, to it, collecting in our way such things as we can glean from out of the mass of words and systematic subdivisions with which this volume is overwhelmed.

Metro-hemorrhagia (we adopt M. Gendrin's learned barbarisms to save time) has always precursory symptoms, which in the main are the same as those marking the catamenial flux: weight in the loins, headach, general uneasiness, nervous agitation, obtuse colicky pains, and, occasionally, diarrhœa. The intensity of these symptoms differs in different individuals. There is no difficulty in recognizing the case in persons who ought to have no discharge, for the existence of the hemorrhage points to the malady; but when the patient can menstruate, the diagnosis between the natural and the morbid flux is often difficult. The discharge is the effect of disease if it be too rapid or too prolonged, *provided the constitution is injuriously affected by it.*

Metro-hemorrhage is a very common complication of the puerperal state. The following is M. Gendrin's account of the lochial discharge:

> "It is not furnished solely by the uterine vessels disengaged from the placenta, and influenced by time and the condition of the uterus to contraction. A portion of the fluid arises from slight lacerations of the cervix. A few hours after labour the discharge is slight, and though constant yet in gushes accompanied by the painful discharge of clots. On the second or third day the exudation is serous mixed with mucosities, which become more and more abundant, taking on at last the sanious character of an inflammatory action which never fails to exist in the uterus after labour. When the "milk-fever" occurs, the last traces of hemorrhage cease, and no more is seen till the establishment of the menses." (p. 92.)

This is not an exact account. The lochia are at first sanguineous; after a few days, generally from three to nine, there is a sero-sanguineous discharge without any mucous admixture, and then it ceases more or less entirely. The order of appearances, namely, first blood then serum, is more constant than the term of duration. The milk-fever, as M. Gendrin calls it, occurs about the third day: it is certainly coincident with the rapid filling of the mammæ; but Cruveilhier, who calls it the "traumatic fever," attributes it to the changes necessary in repairing the wounded uterine surface. The sanious discharge of M. Gendrin is not a phenomenon of health but of disease; where it takes place the layer of new skin applied to the placental spot is decomposing. When pus exudes—and we have seen it exude in large quantities and in its most "laudable" form—the uterine wound is healing by the process of suppuration and granulation. The history of these changes is important, inasmuch as they throw much light on the mode of origin of puerperal fever.

The lochial discharge may readily be converted into a metro-hemorrhage: it may be looked on as a malady if after the lochia have assumed the serous character they take on the sanguineous. During lactation no discharge of blood from the uterus is usual; when it occurs it produces an effect on two constitutions, that of the nurse and nurseling. The former is affected with the general precursors of menstruation; the latter is purged, or there is colic, or there is convulsion. The fact is, that the milk is altered in quantity and quality, becoming scanty, thin, and serous. In those who do not nurse, the regular period occurs about twenty-eight days after the day of actual parturition; and in the weakly, the luxurious, and plethoric, the uterine state favours a profuse flow. If this be not medicated, the foundations of prolapsus and even of malignant disease arise out of the conditions of a congested uterine texture.

The course of metro-hemorrhage is either acute or chronic; when acute, whatever be the epoch, whether after parturition or during the catamenial flux, it is a malady grave in its existence and in its consequences. It may be termed chronic when it begins in a less stormy way but increases progressively until it amounts to a flooding. Sometimes there is a mixture of the acute with the chronic flux, the former appearing as an exacerbation of the latter. With these local appearances there are two sets of symptoms, one attending the discharge in the form of weight in the loins, feverishness, headach, disordered digestion, &c.; in the severer cases, fainting and impending death: the other the result of mere loss of blood, so well described by Dr. Marshall Hall. M. Gendrin has not traced the connexion between menorrhagia and the alterations induced by it in the uterus itself, an omission of great moment, and one we cannot in our limits supply: a glance at the subject might mislead; a detail would be misplaced. Suffice it to say that whenever there is long persistent uterine flux the practitioner should examine the womb, and he will usually find in the softening of the cervix and enlargement of the fundus uteri an organic cause for such persistence.

Neither has M. Gendrin alluded to a form of chlorosis which, allied as it is to purpura, is a source of hemorrhage. At page 107, however, he has noticed the enlargement of the uterus and its occasional ante-version, a displacement, by the way, which in a slight degree is very common; and he has also hinted at the connexion between hemorrhage and organic disease of the womb.

M. Gendrin asserts that metro-hemorrhage not only is a cause of sterility but that if conception occurs abortion will follow: an observation with which we fortify our own experience.

The qualities of the blood, according to our author, change as the disease proceeds. At first the fluid is rich in fibrine, but as more is lost it becomes serous and incoagulable. Sometimes the discharge is completely serous. It occurs generally in chronic flux; nevertheless, we have known it, with great astonishment, assume all the characteristics of a severe flooding, and the woman perish. We have also repeatedly observed what M. Gendrin affirms, that it is accompanied with all the symptoms and consequences of the catamenial discharge.

With regard to the *etiology* of metro-hemorrhage, M. Gendrin says it is but a modification of menstruation, owing to an increase in the intensity of action of these causes which produce the natural monthly flux. As a proof he quotes the known facts, that neither in the period before the age of puberty nor that after that, termed critical, is hemorrhage common; that, further, the symptoms of the natural and diseased discharge coincide, as do likewise the period of their appearance. Another predisponent cause is in the uterine texture, filled as it is with a meshwork of veins. Its situation and anatomical connexions expose it to mechanical shocks, while its functions subject it to excitement, which at once calls into most powerful action both its nervous and vascular systems. M. Gendrin notices the sanguine as well as the nervous temperament as predisposing causes; he remarks, too, that it is hereditary. "We know a family in which all the daughters have, for three generations, been attacked with hemorrhage between the sixth and eighth years of age, one alone excepted; she had repeated epistaxis." (p. 113.) The presence of the malady becomes a cause of its ready reproduction at a future time. Indeed, both the quality of the blood itself and the condition of the vessels altered by hemorrhage are favorable to sanguineous exudation. Besides these causes, M. Gendrin has noted with systematic prolixity those depending on the *acta, circumfusa, applicata, ingesta, animi pathemata, pathologica;* and under the last head he mentions that biliary disease will often produce menorrhagia.

With equal minuteness the prognosis and diagnosis are detailed. The description of the malady would have been benefited by a transposition of many of the paragraphs inserted here; while these two heads of the subject would have gained in vivacity of thought and compactness of matter what they lost in bulk and the ostentatious display of "common place" in the husk of philosophy.

The therapeutics of metro-hemorrhage are founded by M. Gendrin on no less than eight considerations; and these eight are put forth in a style of stilted pedantry which would have annihilated Jean Jacques Rousseau or Voltaire, or any of those authors who have made the French the most transparent medium of thought which now exists. M. Gendrin has completely smothered his native language in barbarisms; a vice which we would not notice did we not find our own mother-tongue be-Frenchified and be-Germanized by a systematic petty larceny at second-hand. Those who know the influence of words on thought will feel it a duty to resist the inundation of expressions which in our modern medical works muddle the pure stream of English and obscure the intellectual treasure it conveys. It is difficult to reject what has now unfortunately been naturalized among us; but we may still stop short of excesses like those of M. Gendrin's—an example of which from the first two indications just alluded to we present: "Le molimen hæmorrhagique qui s'accomplit sur l'uterus pour l'accomplissement physiologique de la menstruation." In this sentence there is scarcely a word of French. It would be unintelligible to the non-medical portion of his countrymen, and not very clear to the student, since two theories (molimen hæmorrhagique and accomplissement physiologique,) are indicated, neither of which are elementary and perhaps not definite. The opening sentence of the next paragraph begins with " L'action stimulante que les causes proégumènes et occasionelles des metro-hæmorrhagies," and is followed up by a thick interspersion of Greek among a few half-starved French words.

However (to return from the form to the matter of our author's work), there are eight considerations derived from the causes enumerated. We have to appreciate in any given case the influence: 1, of the " molimen hæmorrhagicum," i. e. the effort of the constitution to produce the flux; 2, the occasional causes; 3, the uterine structure; 4, the habit of body as to plethora; 5, the degree of uterine congestion at the commencement of and during the menstrual discharge; 6, the facility with which the vascular system of the uterus lapses into passive congestion; 7, the immediate effects of the discharge in giving rise to bloodlessness or hysteralgia; 8, the modifications induced in all these circumstances by age.

The first indication is to withdraw the patient from all circumstances which are favorable to the production of hemorrhage, by enforcing the horizontal posture, by moderating excess in temperature and in nervous action, by freeing the circulation from mechanical pressure, by attention to diet and the due regulation of the bowels; these means, constituting the method of " expectant medicine," are alone sufficient for cure in the minor examples of this malady.

In more grave cases, where the subject is plethoric, venesection must be resorted to in quantity sufficient to destroy the fulness of the vessels. In those who are feeble in constitution, small bleedings repeated at intervals of a few days, while they do not disturb the forces, eradicate the evil. And M. Gendrin recommends most justly the abstraction of blood to be made in all cases just before the menstrual period. Where venesection is useless or inadmissible, leeches should be applied to the neighbourhood of the vulva, either to the hypogaster, the loins, or the anus, especially if the patient is subject to piles. In addition, M. Gendrin recommends derivatives to portions of the body having no direct vascular connexion with the uterus as stimulants to the arms and shoulders. " Menstruas si velis sistere, cucurbitulam quam maximam ad mammas appone," says Hippocrates. M. Gendrin uses dry cupping, by applying two glasses for twenty minutes every hour, beginning at the chest and carrying them down the arm and to the hypogastrium.

These means may be furthered by such agents as quell the local uterine action, as cold water applied to the pubes and vulva or injected into the rectum. M. Gendrin objects to the use of the plug except in severe cases, inasmuch as the mechanical irritation keeps up the vascular. In the chronic form of this malady the indication should be long persisted in. Venesection in very small quantities may be required, but the strength of the patient is especially to be regarded. Where the disease has produced much feebleness from loss of blood, while the patient neglects none of the precautions of the expectant method, tonics must be boldly given, steel, bitters containing tannin, astringents, the mineral waters of Tonbridge Spa, &c.; and if the practitioner sees fit, he may then resort to the nostrums which M. Gendrin has signalized, as cinnamon, sabine, the bark of the green orange, &c. We ourselves place much reliance on the ergot of rye and on vegetable acids. Purgatives of the aloetic kind are proscribed, and justly, by M. Gendrin; but he takes no notice of those of the saline kind, as sulph. magnesiæ, than which nothing is more useful if given three or four days consecutively just before the expected period. Opium in the menorrhagia of nervous subjects is a great remedy.

Utero-Placental Hemorrhage. This is the term used by our author to designate that uterine flux which occurs or may occur during the period of childbearing and childbirth. The whole subject is carefully considered, and some points brought out in a more salient position than is usually met with in books; still the vices of composition, apparent in the preceding chapters, are not omitted here. There is the same prolixity of detail and a similar intricacy of method, the former exhausting all patience, the latter destroying all simplicity and clearness. We shall cull the facts for the reader just as they occur, page by page, utterly despairing of bringing those which would serve mutually to illustrate each other into one point of view.

According to M. Gendrin, the physiological conditions of the uterus during gestation are favorable to flooding, inasmuch as there is not only a natural congestion but also a natural hemorrhage in the interchange of the molecules of blood between the maternal and fœtal systems. Hence the frequency and the importance of the accident. The symptoms and the results on the frame are always of the same kind in every species of "utero-placental" discharge; but there are great differences as to the mode of manifestation and the succession of the phenomena in each—varieties which hinge chiefly on the fact as to the connexion between the ovum and the uterus. This connexion may either be normal or abnormal: the former when the placenta is attached to any portion of the uterine walls but the cervix, the latter when the placenta is implanted on the cervix.

A. Hemorrhage with Normal Implantation of the Placenta. This occurs most frequently before the twenty-fourth week of gestation, and generally coincides with the epoch at which the woman would have menstruated had she not been pregnant. This last remark of M. Gendrin is fully borne out in our own experience, and is strongly recommended as a basis of action. The precursory symptoms of discharge are those of uterine "hyperæmia," heat, fulness, and spasm of the womb, and sympathetic irritation of the bladder, kidneys, and rectum. The development of the muscular fibre of the uterus lays the foundation of spasm of that organ, and deceives the woman into a belief that the motion felt internally is attributable to the movements of the child, which, as M. Gendrin justly observes, is from its gelatinous state incapable of such action. These spasms soon become painful, and are accompanied by erratic flushes of heat and cold. After a few days the flux of blood is ushered in by weight of the loins and small of the back, feebleness, and fever. In severer forms there is great inequality of the circulation and even syncope; and usually in advanced pregnancy inordinate movement of the child. The blood may appear either externally or be concealed within the womb. This is a most important distinction as to practice.

1. *In external or open hemorrhage* the blood may flow continuously or in interrupted jets, accompanied by grinding pain in the hypogaster and loins. With these pains an early ovum is rapidly expelled; but a longer period is required in a more advanced state of pregnancy. To this general description we would add a practical point for the junior practitioner: if there be flooding alone, or pain alternating with ease alone, abortion does not necessarily take place; but if there be both discharge and intermittent pain, the chances are that the ovum will be expelled. It is always to be wished that the whole ovum should be expelled at once in early gestation. A small portion of placenta or membrane left in the uterus will excite a flooding which, even at the third month, we have in more instances than one known as deeply affecting the actual and the future health of the sufferer. If the hemorrhage does not cause expulsion of the ovum, it may still determine its death; a knowledge of which fact we can acquire with greater certainty in proportion to the advanced state of gestation. At all periods, however, there are symptoms the presence of which lead to the inference of suspended gestation: these are—1, the disappearance of those symptoms which had hitherto accompanied pregnancy, such as sickness, &c.; 2, cessation of development in the breasts and abdomen; 3, irritative presence of fever, marked by a feeble, hurried, unequal pulse, sunken features, tarnished skin, disordered hepatic function, and diarrhœa; 4, the uterus is flabby, the fœtus motionless, and not easily moved by the impulse of "ballottement." These cases are very troublesome, from the perpetual anxieties they cause both to patient and practitioner. They rarely go to the full time, yet occasionally they do. The labour is not, however, more liable to hemorrhage, although the puerperal state is to its worst fever, than where the abortion takes place at once. The worst examples, however, in our own experience occur in twin cases, where one of the children has been blighted some weeks previous to birth.

2. *Latent or internal hemorrhage* during gestation is much rarer than external or open flooding. The author speaks of its occurring frequently in the early months of pregnancy; and the general tenor of his context would lead us to believe that it then was similar in its march and effects to internal hemorrhage in the later months of childbearing. Such certainly is not the fact. The uterus before the sixth month is rigid, indistensible, and incapable of holding so much blood as to endanger the life of the patient; and very rarely or never, but in the extremely feeble constitution, does the latent hemorrhage cause the specific danger of an internal and concealed flooding. In a more limited sense, however, we agree with M. Gendrin when he classes under latent hemorrhage those effusions of blood which take place in the placenta or the uterine membranes, giving rise to subsequent abortion and the death of the fœtus. These are similar to those effusions into the lungs of adults known as pulmonary apoplexy, and affect the immature organism by means analogous to the process of destruction in the mature one, viz. in speedily destroying life by impeding the vital changes of the blood.

The symptoms of latent utero-placental hemorrhage shall be given in the author's own words:

"The primary symptoms of latent hemorrhage are precisely the same as those characterizing the open flux; nevertheless the lumbar pains are severer and seem to threaten the expulsion of the ovum. There is at the same time a sense of general discomfort, feebleness, cold sweats bedewing the temples, chilliness, and such a tendency to fainting that the patient often is thrown into a continuous state of 'demi-syncope,' in the midst of which nausea or vomiting produces complete prostration. These symptoms are variable as to duration, but they rarely last longer than four or five hours, and still more seldom do they flow in one uninterrupted course of increasing intensity, but are broken by intervals of remission, during which the patient suffers only from debility and general discomfort. The face, however, becomes paler and the pulse more feeble." (p. 172.)

The symptoms are often less urgent, and recur at intervals of six or eight hours, each paroxysm being preceded by a repetition of those lumbar pains, those tremors, &c., which formed the precursory signs of the first attack. If the blood extravasated into the womb be in a certain quantity, it may become sensible to the external touch. Levret, J. Hopff, Leroux of Dijon, Baudelocque, have severally felt an accessory tumour jutting out the uterine walls.

M. Gendrin notices the union of sensible and latent hemorrhage, under the designation of "demi-latent," for the purpose of stating that the expulsion of the ovum is generally in such cases certain, and accompanied, during the whole process, with a discharge, entailing on the constitution the effects of an abundant flooding.

If, in spite of these discharges, pregnancy persists, the fœtus may remain in utero, as a foreign body, causing a valetudinary state of health, characterized by symptoms of incessant impending abortion: or there may be a gradual increase of the volume of the uterus, dependent on a chronic latent hemorrhage, when all the rational signs of pregnancy have ceased with the blighting of the fœtus. In these instances, the limbs become anasarcous, the digestion difficult, the body enfeebled and meager, the bowels relaxed, the urine scanty, and, finally, hectic fever sets in with a tendency to fainting. This state of things may endure up to the full period of gestation, bringing the patient to the brink of the grave by their exhausting influences. The placenta is found to be, in every instance, larger than in natural labours, its increased bulk being dependent on blood which has become imbedded and entangled in its cellular texture; and with this mass a large quantity of grumous fluid is usually expelled. These labours are less subject to subsequent hemorrhage than any others, a fact quite accordant with our own experience, and one explicable by that *chronic disruption* of the utero-placental bonds, which affords sufficient time for the vessels of the womb to retract and become obliterated.

There are cases of a fatal termination to latent hemorrhage. Some of these M. Gendrin has narrated, and they are well worth attention; indeed the whole subject of latent or, as we term it, internal hemorrhage, is not sufficiently known in all its bearings in this country, although it is by far the most difficult and appalling of the more serious complications of gestation.

"A woman who had remained in the clinic of a private teacher two days was brought," says M. Gendrin, "to our hospital, for pains which seemed to announce labour. She was in her seventh month of pregnancy, and hitherto had not experienced any untoward symptom. The pains were slight and occurring at distant intervals. In order to hasten birth, ergot was given, but without effect. On the second day, all the symptoms of loss of blood being present, she was brought from the private clinic to the hospital. The abdomen was soft, the uterus reached to the epigastrium, its os was closed, and neither the beat of the fœtal heart nor the placental murmur could be heard with the stethoscope. The patient expired in two hours. The ovum was found enveloped in a mass of blood, partially coagulated. The placenta had been torn from its uterine connexions by coagula, which covered two thirds of the upper part of the chorion. A zone, of about three inches in breadth, of adherent chorion formed a dam between the clots and the uterine orifice. The placenta was infiltrated with blood. The liquor amnii was slightly red. The fœtus appeared six months old, and exhibited no trace of decomposition. The umbilical vessels, veins, and arteries were distended with coagulated blood." (p. 180.)

In another instance Albinus found the central portion of the placenta pouched out with blood, which had nevertheless not destroyed its marginal uterine adhesion. We are acquainted with an example in which the patient was seized at the seventh month of pregnancy with constant faintings and all the symptoms of internal hemorrhage. Delivery was forcibly produced, and the amnion was found filled with coagula. In our own experience these dangerous forms of latent hemorrhage, prior to the ninth month, are rare. They are seldom wholly internal in early gestation; seldom, therefore, do they put the practitioner in doubt as to the course of action requisite to save his patient. Where the flooding is concealed, it is of the greatest moment to arrive speedily at a knowledge of the fact. But how is this to be done amid the tumult of anxieties which, in these disastrous examples, spring up in the lying-in chambers to perplex the practitioner? We have been accustomed to watch with extreme care the state of the respiration; for unless the gush be at once immense and sudden (a rare case) the lungs exhibit the effect of flooding, before the brain gives way or the pulse alters much. The long-drawn frequent sigh, the repeated indraughts of air which take place in incessant yawning, prove that the balance between the lungs and the heart is disturbed, and this alone has often induced us to linger at the bedside to ward off a hemorrhage which we knew by these signs had already begun. And let it be remembered that these early moments are those on which health and life hang. The uterus is yet full of contractile power, and readily reacts now; while a few enlarged gushes, unrestrained, exhausting irritability, render hemorrhage a cause of additional flooding.

Among the earliest symptoms, then, of loss of blood is some unusual state of respiration; and in an interval, (whose length is modified by the velocity of the flux, its quantity, and by the previous constitutional state of the patient,) the pulse begins to falter, and the brain to become dizzy with faintness.

3. *Flooding during labour.* The remarks and details of the author are here neither many, important, nor new. In one form of this complication the blood issues externally, and the labour is not suspended. The placenta, on separation, is found to have an adherent clot attached to it, thus pointing out the spot of disruption. In a case of Mauriceau's, the coagulum was of the size of two fists. In other examples the labour is suspended by the grave effects of the loss of blood. In a third form, the flow ceases to be visible; but, nevertheless, is accumulated within the womb, which first does not contract, and then becomes soft, distended, and flaccid, amid all the signs of hemorrhage already noticed. In all these varieties of flooding, the infant may be affected; and in some, respiration after birth is established but slowly, unless the labour shall have been rapid. In proportion to the advance of gestation is the danger of the effects of flooding. It is rarely fatal before the sixth month. Many, however, remain long enfeebled when the drain has been excessive; the slightest disorders become serious, while some, says M. Gendrin, become prone to uterine congestions, menorrhagia, and to its consequences, frequent abortions.

B. Hemorrhage, with Abnormal Implantation of the Placenta. M. Gendrin has the following observation in the very second paragraph of this chapter: "So long as the dilatation of the uterus is confined to its body, that is, from the first to the fourth month of gestation, abnormal implantation of the placenta is unattended by any accident." Now, admitting, with a few authorities, that even from the commencement of pregnancy there is something like a placenta, still the traces are such mere sketches, that the majority of observers have always stated that this organ is non-existent till the third month: hence it is not the want of dilatation of the cervix uteri previous to the fourth month which can be said to prevent flooding. The period at which, in our own experience, we have most often noted its commencement, is after the seventh month. We have never known it as early as M. Gendrin says, viz. after the fourth. In a few instances we can corroborate his statement of the flooding beginning only with labour at the full time. We can also bear our testimony to the fact, that the placenta is not implanted, centre for centre, over the os, in the majority of cases. A lobule, or a portion somewhat larger, edges over the funnel-shaped cervix uteri. The two most remarkable signs which we would notice are the existence of the first gushes, unattended by uterine pain, and their occurrence independent of any assignable mechanical cause. In several instances the flooding has occurred at night when the patient was asleep. The subsequent course of placental hemorrhage is marked by intermissions of a fortnight, then a week, and ultimately by incessant slight discharge, with occasional slight gushes. The fœtus is always in jeopardy and often is killed; but we have no experience as to the assertion of M. Gendrin, (p. 193,) that the flooding ceases as soon as the child is dead, and that labour takes place after this event: we, however, doubt its justness.

Besides the indication furnished by hemorrhage occurring at shortening intervals, without apparent cause, during the latter months of gestation, the os uteri does not, previous to real labour-pains, differ much, judging by the touch, from the natural state. It is perhaps more "*cushiony*," but the head of the fœtus in not felt; still this negative sign may be the result of some other presentation of the fœtus. With labour-pain, however, the case is cleared up; the dilatation thence induced permits us to feel the spongy placenta, and to become sensible of the cause, which, indeed, was readily to be inferred from the gushes of blood coinciding with uterine contraction, and ceasing in part with uterine relaxation. Fortunately, uterine contraction in these miserable cases is almost invariably slow, while the dilatability of the lower segment of the viscus is usually facile. No one need be reminded of the extreme danger, both to mother and infant, in these cases. But there are others where, although the placenta be abnormally implanted, still the hemorrhage ceases and labour proceeds naturally. The fact noted by M. Gendrin we have also observed, viz., that all the signs of "placenta prævia" may have existed during the latter portion of gestation, and yet the labour terminates quite naturally. A portion of the placenta has become detached, and a reparative process has closed up the open-mouthed vessels so as to preclude further bleeding: such is M. Gendrin's explanation in the cases to which we allude. We have found the cessation of the flux to have been dependent, during labour, on pressure of the child's head on the flap of placenta. Wigand (Geburt. des Menschen) has given us six or eight examples of genuine placenta presentations treated by plugging, in every one of which the labour was effected naturally, and without any the least dangerous hemorrhage.

The following extract is a recapitulation of the appearances produced by hemorrhage on the ovum:

"1. If the hemorrhage be slight, one or more deposits will have taken place; the placenta, preserving its natural texture and development around them.

"2. If the hemorrhage be so considerable as for the blood to have become infiltrated into its whole substance, disorganizing it, this viscus and the fœtus are converted into a foreign body. The latter being killed becomes atrophied, dry, and diminished in size, and suspended in a mass of stratified blood. Sometimes the internal flux continues slowly, in which case the placentary mass enlarges, by addition of fluid, and ultimately is expelled in the form of a spongy body, penetrated and surrounded by a great quantity of liquid blood.

"3. If the hemorrhage be rapid, coagula surround the ovum, and sometimes lacerate the placenta and membranes, and in a short time the mass is expelled." (p. 210.)

The quantity of blood which may be poured out on the centre of the placenta without detaching its edges from the uterus, we have seen, in the case of Albinus, to have been sufficient to cause death. M. Gendrin quotes another remarkable case of the same kind:

"A woman, thirty-six years of age, the mother of many children, and in her eighth month of pregnancy, had a violent cough and fever. Being seized with labour she sent for the midwife, who, after twelve hours of travail, saw her patient fall into the most alarming state. M. Delaforterie was sent for, but arrived only after her death. He instantly performed the Cæsarian section with every requisite precaution; and on opening the fundus uteri witnessed the escape of a gush of blood, which he estimated at three 'chopines' at least. This fluid left a large pouch between the placenta and uterus, into which M. Delaforterie, on introducing his hand, was satisfied that the placenta had preserved its natural marginal adhesions with the womb. The fundus of the latter organ exhibited no trace of rupture. There was no blood in the vagina; the uterine orifice was little dilated. The child, though extracted alive, lived only a few seconds." (p. 211.)

The extent, however, of this extravasation, must be considered as remarkable as its mode of inclusion. Usually, says M. Gendrin, the clots imbedded in the placenta are small, seldom exceeding in bulk a pullet's egg; oftener they are smaller, and interspersed over the placental surface. They are of a yellowish-gray colour, solid, and, as it were, fleshy; they are, he adds, very common, and would have been oftener noticed were not the examination of the placenta habitually neglected. Their smallness accounts for the safety of the mother, while their presence will explain the debility of the fœtus in many instances, and the atrophied state of the organ, at once impeded in function and restrained in bulk. We would strongly recommend these observations to the reader. Nine tenths of the abortions are produced by the effects of a disordered utero-placental circulation. We believe, however, that the yellowish-gray masses, described by Gendrin, are, in some instances at least, a disease of the placenta itself, independent of effusion. Such placentæ we have repeatedly examined, and found all those changes going on which are visible in the conversion of cellular membrane into bone: in one part, perhaps, a little thickening, in another a yellowish cartilaginous texture; in a third spiculæ, which feel like bony texture. We need not add that in such examples the after-birth has been adherent, and the child ill-nourished.

In these effusions of blood into the placenta, M. Gendrin remarks, that the liquor amnii is usually reddish; and the infant what is termed blighted; and though retained in utero, its arrestation or death is usually coincident with the first symptom of hemorrhage. These observations tally with our own experience.

With regard to the lesions observable in the placenta where that organ has been implanted on the os uteri, M. Gendrin has dissected, since the year 1824, forty-two such examples. The following observations merit great attention; we must, however, abridge them:

"If the placenta is implanted only in a small extent over the os uteri, the portion so engaged is as friable as the spleen, but looks like the lungs when thrust by pleuritic effusion against the vertebral column. The spongy tissue is obliterated and rendered homogeneous by blood incorporated with it. This, the simplest alteration, is observed only when the hemorrhage just precedes or even accompanies the expulsion. In cases where the flooding has occurred at distant intervals, there is a larger segment of placenta implanted, and the alterations are various in three points, viz. 1, the centre; 2, the intermediate zone; and 3, the outer margin of the organ. The central portion is condensed, homogeneous, granular, grayish-yellow in colour, readily friable, and intersected by white filaments, which extend in ramifications to the uterine surface, but are lost in the yellow layer; which does not present, throughout its thickness, a single point of redness. In the midst of the homogeneous tissue small reddish-black clots are seen, usually in great numbers, not separable from, but confounded with its substance. These small clots ordinarily penetrate to the chorion. The surface of the placenta, in these altered portions, presents white spots of a few lines diameter, having the aspect of those tuberculous deposits found on the peritoneum. The intermediate zone of placenta is red, with coagulated blood infiltrated into, and incorporated with its texture. It is much softer and more friable than in the healthy organ. Its homogeneous appearance is broken up by cysts of blood of varying depths. The exterior zone has the same aspect as that described as exhibited in partial implantation of the placenta." (p. 217.)

From this description M. Gendrin concludes that the lesions mark the epochs of hemorrhage, the central zone being the result of the earliest. The other lesions depend on ruptures produced either by the uterine expulsive efforts, or those incident to extraction by the accoucheur.

M. Gendrin ascribes the hemorrhage "unavoidable," as it is termed, not to separation but to rupture of the placenta from the uterus. The alterations into the yellowish-gray matter just noticed is a process of reparation by means of which, as already observed, the flooding of the earlier months is stayed during the latter; and the labour, though a placental one, is not necessarily hemorrhagic.

*Causes of utero-placental hemorrhage.* Pregnancy, by establishing a natural congestion in the uterus, must of course be regarded as the predisposing cause of the flooding which often attends it. Menstruation, too, or the habit thence induced, is another; and we have before assented to M. Gendrin's remark, that the floodings of gestation coincide, for the most part, with what would have been in the unimpregnated state a menstrual period. Besides these, there are certain temperaments which predispose to uterine bleeding: 1. The sanguine: marked by a powerful circulating apparatus. 2. The lymphatic: characterized by a languid transmission of the blood by means of a feeble heart and arterial system, by a great development, on the other hand, of the capillaries, and by a large though feeble frame. 3. The nervous: fragile in form, pale in aspect, but full of pains which flit from organ to organ: these women, for the most part, are subject to irregular uterine gushes, from extreme uterine irritability.

Mechanical causes acting on the uterus, it is well known, will produce flooding; but there must be a predisposition to it, judging from the numerous examples in which the concussion does not end in discharge. Nevertheless, Smellie has noted this result as succeeding violent vomiting; Van Swieten, sneezing; and others have remarked a similar effect as resulting from the spasms of an hysteric fit.

Moral impressions of a violent or intense kind, whether exciting or depressing, doubtless must be accounted as frequent and effective occasional causes.

M. Gendrin notes the disappearance of varices from the limbs as inducing uterine discharge; and also remarks the tendency of hot, hip, or foot baths, drastic purges, bleeding in the foot, &c. as producing flooding.

Our author next passes to the consideration of diseases of the uterus as causes of hemorrhage. He relates that it occurred once after inflammation, once where fibrous tumours were contained in the uterine cavity, and in a third case where the cervix uteri was much enlarged; but the most frequent of this class of causes, he thinks, is inflammation of the ovaria and Fallopian tubes: indeed he would connect this state of these organs and uterine flooding almost as a constant conjunction. We ourselves have found that "the elongated cervix" is both accompanied by menorrhagia in the unimpregnated and flooding in those who have become so in a few instances.

The influence of polypus has been very marked in the two examples where it existed as a complication of labour—though in a third case, in which the polypus was of great size, no flooding took place. We have known several examples in the practice of others of very large fibrous tumours not causing inordinate discharge. As to inflammation of the ovaria, Boivin attributes one class of abortions to the adhesions formed by the Fallopian tubes hindering uterine expansion. We have not remarked the coincidence between flooding and inflammation of these organs, or perhaps we have overlooked it. There are many physiological facts, however, which would incline us to receive it, if not with all the exclusive faith of our author, yet with much.

The anormal insertion of the placenta is, according to our author, a very frequent cause of uterine hemorrhage. Madame La Chapelle says it is the sole cause of flooding in the latter months of gestation. Rigby, of Norwich, found, in 106 of this last class, 46 resulting from the anormal implantation of the placenta near the os. According to M. Gendrin, unless this position of the placenta be very obvious, it is usually overlooked by accoucheurs.

*Proximate causes.* The early hemorrhages have for their proximate cause the disruption of the vascular connexion between the decidua and womb. Both deciduæ, the true and the reflected, are, says M. Gendrin, vascular. In spite of M. Velpeau, we too adopt this view, and have tried the experiment of W. Hunter of spreading a piece of these membranes on a sheet of paper and holding it to the fire, to show us, in its black streaks, that blood coagulated which had flowed in its very thin vessels. These very early hemorrhages are not unfrequently thought to be a retarded menstruation bursting out with more than ordinary violence precisely because of the retardation. If they occur in married women previously quite regular, or if a woman has been regular both as to time and quantity of discharge two or three consecutive months, and then there follows a retardation as to time and an increase as to quantity, it is advisable to treat such persons as having suffered early miscarriage, in order to give them a chance of gestation as well as of conception.

M. Gendrin gives us three ingenious conjectures as to the causes of the anomalous implantation of the placenta: 1. The orifice of the Fallopian tubes may open nearer the os than the fundus uteri—a fact observed by him in the dead body; he nevertheless does not attach much weight to his own conjecture, neither do we. 2. The ovum may glide too low. 3. The thick mucus contained in the Fallopian tubes may project too much behind the decidua, and so permit the ovule to descend.

From the second to about the twelfth to the fourteenth week, we know that the deciduæ are approaching each other until they coalesce at the last-named period. During this time the circulation in the maternal vessels of these membranes is ample: hence any of those causes which act in the production of menorrhagia cannot fail to influence the chances of uterine hemorrhage at this point of gestation.

From the twelfth week a double vascular organization is going on, in the formation of a fœtal and a maternal placenta. On the maternal side the decidua serotina is thicker and more vascular than any other part of the caduca lining the uterus, and on the fetal side the umbilical vessels have begun to climb into the villi of the chorion, to form many tortuosities, and twistings, and knobs. In this way the two systems of circulation mutually interproject, without ever commingling directly their several fluids. This early type of the placenta is the permanent formation in the horse, in which animal we have repeatedly seen that the chorion, vascular in all its surface, is in juxta-position merely with the vessels of the lining membrane of the uterus; that its vascularity is equally diffused, and that the union between them is just what may be represented by the apposition of two wet cloths.

At the middle period of gestation in the human female the maternal placenta is more than a third of an inch thick. Its vessels have two peculiarities: they are first very thin—so thin as to be, according to some, mere prolongations of the inner membrane of the uterine veins. According to Stanley and Mayo, the column of maternal blood is carried into canals formed by thin projections of the decidua. Whatever opinion be adopted, this is true—that the decidual vessels are very thin. The second peculiarity is that they expand into pouches at their extremities. The circle formed by the line at which the decidua vera becomes reflected into the decidua reflexa, constitutes the limits of the placental discs; here the union with the uterus is most intimate. This collar, conterminous with the edges of the placenta, is more solid in the earlier months; hence the known difficulty of abstracting the placenta in abortion. It becomes less intricate as gestation advances; hence the comparative facility of removing this organ. In the early periods of placental formation, the maternal disc is as thick as—M. Gendrin says thicker than—the fœtal, but gradually becomes thinner towards the latter end of gestation; from which circumstance he would deduce the cause why the fœtus is more independent of hemorrhage in the end than at the beginning of pregnancy.

*Uterine turgescence* is next viewed as a proximate cause of flooding, or as M. Gendrin calls it in his margin, "cause immédiate." Now, though there cannot be two immediate or proximate causes of any effect, still we may look on the expression as indicating a condition without which flooding could not exist—the "causa sine qua non" of logicians.

The first change determined by conception is afflux of blood into the whole generative apparatus from the ovary to the os tincæ; and this afflux continues with such unceasing vigour that in the last months of gestation the uterus may be looked on as an erectile tissue, between the internal and external strata of muscular fibre of which a layer of thick, very short, but capacious veins is interposed. These veins are so short as to pass for cells; but they resemble exactly the distribution of the cells of the corpora cavernosa penis, which Tiedemann has proved to be a congeries of veins. The arterial distribution is also copious, and may, at a glance at William Hunter's plates, be thoroughly appreciated. The termination of these veins on the fœtal placenta and the decidua, their size, so expressively named "sinus," their number, friability, present to the student an apparatus for circulation, in any derangement of which he will readily apprehend why the effect should be so terrible as to have deserved, even at the hands of the common observer, the appropriate appellation of a "flooding."

We may fairly then grant that with such a supply of blood, and with such an arrangement, the vascularity of the uterus must be considered as an immediate cause of its hemorrhage. M. Gendrin after giving a very incomplete description of the uterine mucles, seeks in their spasmodic action for another influential agent in hemorrhage. Spasms are irregular contractions; irregular contractions may tear up the placental or decidual organs; hence hemorrhage: such is his position, strengthened by the fact that the patient experiences not unfrequently violent movements which precede the visible or the latent flux. We admit in theory this cause. In practice we have known uterine spasms occur for months daily, in spite of large opiates constantly administered; and rarely, indeed never, have we seen them occasion flooding. The agency of the muscularity of the uterus is especially preservative: these fibres are the natural ligatures to vessels, which without their contraction would sluice away life in a very few seconds: still, however, we admit the theoretical probability of irregular uterine contraction being followed by hemorrhage during gestation. After childbirth, irregular contraction, in its broadest acceptation, combined with partial or total separation of the placenta, is indeed the commonest condition of flooding. We differ with M. Gendrin when he states his disbelief of the uterine spasm, so often noticed by women as "violent motion" of the child, being really in most cases actual fœtal movement: we have, in the severer complications of labour, in convulsions, in hemorrhage, felt this fœtal movement and known it to precede the death of the infant; while, in other examples of flooding, we have had every reason to believe that the flooding *preceded* the movement, which though it certainly existed as uterine, was caused by and did not cause flooding.

*Placenta prævia.* M. Gendrin now proceeds to consider how hemorrhage takes place in placenta prævia, (p. 267;) and the speculations are to us new and curious. It is usually thought that in these cases the necessary expansion of the cervix uteri, in the latter months of gestation, tears away the connexions between the placenta and womb, and thus freeing the uterine sinuses, gives rise to "unavoidable" hemorrhage. M. Gendrin, however, takes just the opposite view, and asserts that the flooding is owing to ruptures, not of the uterine vessels from the edges of the placenta, but of the central parts of this last organ only. According to him, the circle sketched out on the womb by the reflection of the decidua vera into decidua reflexa, is the limit of the edges of the placenta; that this organ never overslips this limit; that the expansion of the womb increases the capacity of this circle without altering the original fixture of the circumference: in a word, that the placenta does not grow by extending its edges, but by increasing its centre in the increased space formed by the expansion of the fundus uteri. Now, if the placenta be fixed, from first to last, to the cervix uteri, as long as the cervix dilates and is accompanied by just as much placental development as will fill the increased uterine space, no hemorrhage will ensue; and this happens during the first half of gestation, when, though the cervix uteri begins very early to expand, still no bleeding occurs. In the latter half of gestation, however, the neck of the uterus does not expand so rapidly as the placenta grows: the result is "that the placentary adhesions are necessarily destroyed from the centre towards the circumference of the cervix. The mucous membrane of this last part is separated from the placenta by a mere layer of mucus; then it is, that there are transudations from the placental vessels. If the placenta be attached by its edges to the cervix uteri, the separation of the sides of the neck breaks up the placental cotyledons; as much by the distension they suffer, as by the endeavour to become engaged in the cavity of the cervix." (p. 269.)

If our paraphrase of M. Gendrin's views be correct, he believes that the bleeding in placenta cases is from ruptures in, or exudations from the placenta almost always, and not from denudations of the uterine sinuses. The anatomical details already given of the state of the placenta in such cases, if exact, would afford some colour to M. Gendrin's speculations. Still we are inclined to retain our former belief, that the source of flooding in placenta prævia, is from this organ being torn by uterine expansion or contraction from the womb, and not from a rupture of the central parts of the placenta itself. It is true that many of the phenomena of these miserable cases will be explained by either theory; that, for example, the gushes attendant on labour-pain, and ceasing when the uterus ceases to contract, may equally exist under a supposition that the placental rent is further lacerated, or the coagula stopping it removed by the expansive action of the os during uterine contraction; as by the old supposition, that this contraction has created fresh separation between the vessels which formed the connexion between the womb and the placenta. Under either theory, we say, the remittent gushes coincident with remittent uterine action are accounted for: but surely the mass of blood lost is maternal, which would scarcely be the case if it came from a rupture of the after-birth: the very tangled and interlaced texture of this organ makes the flux from rupture comparatively slow. We must remember, too, that the placenta is made up in bulk by fœtal vessels, among which the deciduous maternal vessels are placed; hence the escape of any one infant, in ruptured placenta, is, under the theory of M. Gendrin, scarcely possible, and yet a certain number are always saved in placenta presentations. We have delivered both by perforating the placenta in its centre, and by separating its edges, and have found the flux of blood much greater in the latter than in the former cases; so much so, that where the mother had already lost much, we have invariably on the grounds of a less, and a less rapid hemorrhage, preferred perforating the placenta to separating it, in cases where the implantation has been centre to centre.

*Diagnosis of utero-placental hemorrhage.* In all doubtful but important cases, it is necessary to make a thorough examination, and not to hesitate to introduce a part or the whole of the hand if requisite. Utero-placental hemorrhage may be distinguished from menstruation during pregnancy, by the presence of pain in the former, and its absence in the latter; by the periodicity and by the quantity of discharge. Sometimes there is a varicose state of the vagina, which would be liable to mislead those who do not examine the state of the organs themselves.

The diagnosis of placenta prævia, M. Gendrin says, is not so easy by the touch as books would make us believe, and we agree with him. In the first stages of labour, before the os uteri is much open, the kind of hemorrhage has been our guide to suspicion. Clots entangled at the os uteri prevent a due recognition of what is behind them; and mere curiosity must not have place in a case where the removal of a clot may be followed by a fatal gush; still, the absence of the resistant sensation which the fœtal head affords, even through the uterine walls, coupled with the kind of hemorrhage, is generally deficient in the last weeks of pregnancy, to leave few doubts as to the case. When, during early labour, the os uteri is open, the unequal spongy substance of the placenta is readily, we think, known; but M. Gendrin adds a very valuable sign of diagnosis, if it be well made out, viz., that there will be felt a pulsation at the os uteri, not synchronous with that of the mother's pulse, but with the quick measure of the fœtal heart. We have never as yet tried this: indeed, in placental cases, he who takes the earliest period in which it is possible to deliver, will be the safest practitioner, and we rarely could venture to do otherwise. A second mark is the impossibility of producing *ballottement*, which M. Gendrin found in three examples: he, however, does not assign this as positive. We know of another instance corroborative of this diagnostic symptom. An eminent practitioner, now dead, being consulted in a case of doubtful pregnancy, detected every sign but that known under the name of "ballottement:" though, therefore, he could be sure of an enlarged uterus, he could not say positively whether such enlargement was owing to the presence of a fœtus: he gave it as his opinion, that if the woman were pregnant, the placenta was interposed between the cervix uteri and the head of the child. This sagacious surmise eventually proved quite true. Before, however, the ballottement be taken as a test of placenta prævia, and before the practitioner should indulge himself or the friends of his patient, in the gloomy anticipations attendant on such examples, he should be aware that this bounding movement given to the fœtal body floating in liquid, can only be produced with certainty between the five and a half and seven and a half months of gestation. Even then there must also be that adjustment between the developed weight of the fœtus and the quantity of liquor amnii, that the former shall be so nearly of the same specific gravity as the latter, as to be readily made to ascend and descend. If this proportion be not exact, and the fœtus be too heavy, it cannot be moved; and if too light, it will not descend: in either case the ballottement is imperfect or null. The stethoscope gives, we think, in placenta cases, a very doubtful diagnosis. In Dr. West's very neat translation of the younger Naegele's work on Obstetric Auscultation, it is asserted that in placenta prævia the murmur is heard loudest over the pubis. This sign can but be considered as an adjunct to others: it is very deceptive by itself.

The history of the case is, however, the most valuable diagnostic element. "Flooding, without pain, at decreasing intervals prior to labour, and flooding coincident with uterine pain, in labour," will be sufficient grounds for the strongest suspicion.

The diagnosis of internal hemorrhage is most difficult and most important. We have already slightly noticed the extreme value of changes of respiration, sighing or yawning, as indicating, prior to syncope, the presence of concealed flooding. Latent hemorrhage, in the early months of gestation, may be inferred from the following group of symptoms: sudden uterine spasm, a shivering fit, cold extremities followed by syncope actual or impending, accompanied or succeeded by "*tenderness* on pressing the womb." This condition ends by a discharge by the vagina of serum slightly tinged, and by slow recovery from fretful fever; or by a continuance and even aggravation of this fever, with a cessation of abdominal expansion, so as to lead to the suspicion of the death of the fœtus.

In the later periods of gestation when the womb is distensible, the quantity of blood effused being greater produces more marked effects—sudden syncope, or sudden diminution in the force, with increased rapidity of the pulse: then follow the effects of loss of blood—the pallid cheek, hollow eyes, limbs bedewed with cold sweats, hurried respiration, great terror, and great helplessness. If now the hand be laid on the abdomen, the womb will be felt soft, doughy, obscurely fluctuating, flaccid, and *suddenly enlarged.*

Intermittent internal hemorrhage may be suspected by the presence of spasm of the uterus, by more or less of syncope, and the continuance of feeble health, only broken in on by those storms which threaten to sweep away the double life.

Latent hemorrhage, *during* actual labour, is known by the above symptoms of syncope and coldness, followed by the sudden diminution of the pains; by increased uterine bulk during the cessation of uterine contraction, and especially by that diagnostic of Levret, pain on pressing the uterine tumour.

*Treatment.* Our author divides cases of uterine hemorrhage into two classes: in the one those accidents have not as yet occurred which put a period to gestation; in the other, it is impossible to prevent the expulsion of the uterine contents. In the former cases the object of medication is to prevent labour; in the second to hasten it; while, at the same time, every means is resorted to to stop the flooding. In the first class, then, of cases, the first thing to do is to allay every symptom precursive of excessive uterine plethora. We are not to wait till there has actually been a flux before we act; it then is often too late; but our prophylactic treatment should commence with well-grounded suspicions of disordered circulation in the womb. The rules laid down by our author on this head are abundantly strict:

" Abstract even the slightest causes tending to keep up uterine congestion; remove every band which restrains the current of blood; let the patient assume the horizontal posture, from which she is to swerve neither for the wants of nature nor the toilette; the pelvis must be raised above the level of the rest of the body; the temperature of the room should be rather cool than hot; all causes which act directly on the uterus as well as all moral influences should be abstracted; all objects of undue interest and all intellectual occupation must be eschewed; the diet should sustain but not excite, so that the digestive organs should be called into a feeble activity only; the alvine secretions should, however, be sedulously regulated." (p. 306.) Such are M. Gendrin's directions: an excellent mark to aim at certainly; but he who expects to effect such a change in the excitable habits of the upper classes, as to induce them thus to vegetate, ought to furnish us with a recipe to control the ennui which preys on minds which are told to banish all feeling and all intellect. These little exaggerations of M. Gendrin are pardonable, however, and may be even commendable, if rightly appreciated, as including good directions for keeping the body and mind quiet, without attempting to torment the latter while you imprison the former. These means, says M. Gendrin, should be enforced for twelve weeks, when the placenta has a more developed energy and hold. If, however, the flooding continue in the fourth month, the same regimen must be enforced until the last month of gestation.

Bleeding is discussed cautiously by our author. He allows that it is a provocative of abortion injudiciously used, while, on the other hand, it saves the plethoric from this result if timely employed: even in those uterine irritations, the common result of the congestion of pregnancy, he does not scruple to recommend the authority of Mauriceau who, in one case, bled eighty times during gestation. We are certainly one of those whose experience is not favorable to the use of this remedy in gestation. The cases must be well marked indeed which require general bleeding: on the other hand the greatest amount of plethoric ailment in the mass of women with child, yields to a cupping on the loins or to leeching. The nervous shock in the excited state of system induced by pregnancy is very great among the wealthier classes, and cannot be disregarded but at a woful sacrifice. Among the hard-fibred and indifferent, in cases, too, where there is no doubt as to uterine as well as general plethora, venesection may be demanded, but it should never be resorted to unless the indications be most positive. M. Gendrin, however, by stating the dangers of over-bleeding or of injurious bleeding, has neutralized the "verba magistralia" as to its employment. He puts forth prominently the increase of nervousness, the constant headach, the perpetual febricula, the deranged digestion, the uterine spasms reproducing uterine hemorrhage, the subsequent difficulty, not to say impossibility, of combating these "symptoms caused by the lancet" with any hope of success. (p. 312.) To these observations there are appended others indicating the great danger of inducing syncope in pregnant women; the necessity, therefore, of never bleeding but by a small aperture, and in the horizontal position; and, further, of never reiterating the emission save it be ascertained that the process of blood-making is rapid and certain.

With regard to the indications for local bleeding, our author names the constant presence of lumbar pains and the appearance of varices and hemorrhoids. Care must be taken to shun producing syncope, to avoid, also, bleeding when there is no local turgescence, as then the local emission produces, by attraction, local congestion.

*Irritants* are liable to less objections than depletants, according to M. Gendrin: hence he quotes Riverius as recommending sinapisms, dry cupping to the thorax, mammæ, hypochondria, and abdomen. Care should be taken, however, not to over-excite the nervous system; hence irritants rather than vesicants are to be employed.

*Sedatives* are a dominant indication in uterine flux. Having given repose to body and mind, cold should be used; and, first, the drinks should be all cool before they be gradually reduced to the temperature of ice. The solid food should also, according to our author, be cold. Cold, sponging, tepid cataplasms about the abdomen and loins, with the derivative treatment, are among the most successful means of arresting hemorrhage. The cold hip-bath, too, is very useful; but the temperature should be cautiously regulated to the feelings of the patient.

Our author having asserted that ovaritis induces uterine flux, recommends general or topical bleeding when, during pregnancy, these states are coincident; remarking, however, that the disorder of the ovary being kept up by the natural plethora of pregnancy, is rarely removed till the latter months.

If these means do not succeed, M. Gendrin makes an issue ("exutoire profond") at the lower part of the linea alba—a practice which we would by no means imitate nor recommend. The distinguishing the symptoms of a turgid ovary from those incident to a plethoric uterus must be a nice point; the constant coincidence of uterine flux and ovaritis is yet to be proved; the only part certain, then, is the torment of a profound issue over the pubic region, inflicted by a gentleman who, three pages before, is recounting the dangers of simple irritants on the susceptible nervous system of the pregnant female.

There remains one other set of remedies which, added to the abstraction of blood, the use of cold and of revulsives, complete the treatment of the first class of cases, viz. *anti-spasmodics:* of these opiates are the chief. "We are not acquainted," says M. Gendrin, "with any therapeutic agent meriting more confidence than opium, whenever the uterus during gestation becomes the seat of spasmodic pains; nay, we never wait for the appearance of those shocks which give rise to these in pregnancy, and we resort to it whenever even there is an irritable nervous system." (p. 328.) Even when this valuable drug does not arrest it will mitigate the discharge. Smellie resorted to opium in all the serious hemorrhages of gestation, early or late. Twenty or thirty drops of laudanum will immediately arrest, says M. Gendrin, a flow of blood which threatens to throw the patient into alarming debility, portending an immediate fatal termination. In this praise of opium we most cordially join. There is scarcely a case of inordinate flux in which it is not the best remedy, save in actual labour, when it is only second to manual aid, which, let it be emphatically understood, is in these cases the first. In pregnancy the usual dose of opium may be advantageously increased, and its effects should always be sustained by a regular repetition of it. A more direct effect is produced on the uterus if it be administered as a lavement, while the stomach and liver are not disordered as by the usual mode of taking this medicine. M. Gendrin combines opium with syrup of orange peel, extract of quina, sulphate of quina, gaseous alkaline drinks, for the purpose of remedying these effects on the stomach. This practice of Hoffman and Riviere is of singular benefit, and enables the practitioner to continue for months the use of opium with great advantage.

With regard to the second class of cases, those in which gestation is interrupted, the chief indications are as to the means by which the flux is most speedily quelled, all considerations as to the preservation of the gestatory state being banished from our mind. If the child have been killed by the hemorrhage, but labour has not come on, the flux usually ceases; and though we do not state it so peremptorily as M. Gendrin, certainly there is not much hazard of a recurrence of flooding after the death of the fœtus. The labour at a later period is generally unhemorrhagic; hence in these cases the indications are to guide the constitution through that group of morbific influences which we have already described as attending the presence of a dead fœtus in utero. Premature labour should rarely or never be induced in these examples. M. Gendrin says that the woman, whatever may have been the actual loss of blood, need not, after the blight of the fœtus, be kept supine, except (he adds) in those very rare examples of a continuance of the flux. Where the intra-uterine flux has been extreme, inducing extreme debility, the indication is, according to our author, to gain time until the forces of the woman shall have been recruited to withstand the chances of a renewed hemorrhage during labour.

If the hemorrhage take place in the early periods of gestation, the uterus not being capable of containing a quantity detrimental to life, the woman is not in danger so long as the flux is not external; hence, in the early months of pregnancy, those means must be resorted to which restrain flooding within that limit, so that it shall not become external; and we must wait the expulsion of the detached ovum by nature, and never, within these limits of internal hemorrhage in a small womb, must we provoke labour.

If after the fifth month of gestation flooding occurs, the effect is much more decided on the mother. If it be intense, the patient may succumb in a few minutes. These are cases never to be left until the hemorrhage ceases, or until the fœtus is expelled or extracted. When the flooding is external there is not so much chance, says M. Gendrin, of utero-placental lesion as when it is internal: there are examples, and these not rare, where after such a gush the woman has still brought forth at the full period a live child. But he wisely adds that these instances should not lead to the adoption of the expectant method by the hope of similar results to ourselves. The dictum of old Mauriceau should form the rule, a deviation from it the exception: "At what period soever of gestation the flooding exists, whether early or late, the most expedient and most salutary remedy for the safety of the mother and infant is to deliver as quickly as possible. When the blood is so abundant in its flow as to throw the woman into frequent faintings and convulsions, the operation is not to be deferred; it is absolutely necessary to deliver her whether she be at her full time or not, or whether there be pains or not; unless, by neglecting these sole means of safety, you will be content to witness the last wave of blood gush out at the same time with the last sigh."

There remains for the most dangerous case the operation of *forcing* the uterus and turning the child. Forcing is a term, however, rarely realized in English obstetricy. In hemorrhage, the os soon becomes flaccid, and requires no force for its dilatation. In those cases in which the delivery need not be instantaneous, M. Gendrin recommends, in common with most authorities, the rupturing the membranes, a practice first, we believe, adopted by Puzos, who waited for the bulging bag of the amnion before he punctured; while Smellie recommends the operation to be done as soon as possible. M. Gendrin says little or nothing on this very valuable remedial measure. Its merits, however, have been denied by some and exaggerated by others. Let us be distinctly understood, however, that the great—nay very great—majority of cases of flooding *during* labours are successfully treated by this mode of proceeding. Smellie, Baudelocque, Rigby of Norwich, and Merriman, have all given their assent to this practice. We believe that Hamilton of Edinburgh rarely recommended it. Of this, however, we only speak as former listeners to his lectures, the best we ever heard on midwifery. The exaggerators of the efficacy of puncturing the membranes as a remedy always successful have to answer for its indiscriminate use. It is not safe where the flooding is violent; it is not safe where the patient is much enfeebled; it is not safe where the fœtus does not present in its long axis; and

Boivin would add, that it is not safe where there is little liquor amnii and a large child, for the uterus then cannot contract—the sole object for which the membranes are ruptured. In those cases of hemorrhage during labour anterior to the rupture of the membranes, the examination should be made with the whole hand if requisite, and every point thoroughly determined before we proceed to any course of action. It is not always easy to perforate the membranes which in the commencement of labour often fit the child's head like a scalp. There has been a question whether plugging the vagina in cases of the kind now under discussion is not safer than rupturing the membrane. The quantity of blood pent up within the uterus, say the advocates of this mode of proceeding, must necessarily be slight, while the presence of the clots in utero and of the "tampon" in the vagina invariably produces labour-pains. So that while we preserve the membranes entire, we remove all those risks which arise in labours from premature evacuations of the liquor amnii. We confess we never have had the courage to attempt this course of action in similar cases. The reasoning is plausible, nay more, it has been supported by the practice of Baudelocque and others. In cases of placenta prævia, where we cannot dilate the orifice of the uterus, plugging is the only remedy; and it is so efficacious, that Wigand advises us to resort to no other, giving six or eight instances of the child having been born quite by na tural efforts, as guerdon of his success. If plugging should be determined on, the operation should be so exact that not a drop of blood should escape externally. We never quit the bed-side whenever we have been induced to resort to the "tampon," and we would advise the practitioner to attend both to the rational signs of hemorrhage as well as to the sensible one of a *distending* abdomen, and to act accordingly.

We need not, we presume, guard against our meaning being misunderstood, by stating that plugging is only to be resorted to when the uterus will not contain blood enough to injure the mother, consequently it never should be used *after the birth of the child* from the seventh month to the full period. Before the birth of the child, however, and before the rupture of the membranes, the case may occur in which the "tampon" is our sole efficient remedy; and then it is highly probable, though we believe not certain, that the womb will not admit of much further distension without immediate reaction. We say not certain—because we have known the blood burst through the membranes into the amnion, and we have cited examples of its being accumulated in the centre of the placenta. These, however, are rare cases, and ought to make us act with caution while we retain the operation of plugging, not as superseding that of puncturing the membranes, but as suited to peculiar states.

As to the mode of action remedial of the hemorrhage incidental to abnormal implantation of the placenta, the following important observations of M. Gendrin must be stated in his own words:

"Hemorrhage always ceases as soon as expulsive labour-pains commence, in cases where a portion of the placenta only is fixed over the os uteri. (Smellie, t. ii. p. 247 and 356.) The same result takes place where the centre of the placenta is over the os, as soon as the expulsive uterine efforts have ruptured the amnion. Flooding only recurs if there be inertia uteri. We are astonished that this remark has escaped the greater number of accoucheurs. If a gush of blood succeeds each uterine contraction, it is only in the first period of labour, and until the uterine contractions have become thoroughly established, in cases where the placenta is attached only by a segment to the os. When the attachment is centre to centre, then the flux continues up to the bursting of the amnion. This has been the course of matters in every case which we have seen. Hence we always hail the advent of true labour in placenta cases. We would thence also further deduce this point of practice, viz.—the necessity of producing labour artificially whenever the flooding is in quantity or by frequent recurrence likely to injure mother and child." (p. 348.)

This recommendation is precisely what is affected by all accoucheurs, when in these terrible examples of flooding they turn the child; but this, the usual course, is not the one which M. Gendrin advises. He resorts to a method which he has tested by actual experience as capable of saving both mother and child. The plan is, to evacuate the liquor amnii by passing the requisite instrument through the placenta and so puncturing the membranes. M. Gendrin has resorted twice to this operation, and has been twice successful: Let us abstract these instances.

"Case i. A fair young woman had a gush of blood at the sixth month, and three more during the seventh; for each of which she was bled by her attendant and thus reduced by the double loss to a state of debility so extreme as to make the upright posture in bed a cause of fainting. M. Gendrin being consulted, declared the case to be one of anormal implantation of the placenta, and recommended the puncturing the membranes, declaring that another gush of blood would kill her. The original attendant deeming the case one of mere congestion of the os uteri, resolved to plug the vagina, but while he was preparing the 'tampon' a fresh gush took place which at once induced M. Gendrin to introduce his hand into the cavity of the pelvis. He found the cervix uteri rigid, and permitting him only to pass the tip of his finger, high enough however to allow him to recognize the placenta. His original proposition occurred to him of evacuating the waters by puncturing through the placenta. A female catheter —" une algalie ordinaire de femme"—was the instrument used, which, with the greatest facility penetrated the amnion through the intervening parts, and the liquor amnii immediately escaped. The hemorrhage *ceased instantly*; but labour-pains did not take place till three hours after the operation; and in four more a child was born alive, which protruded the placenta before a part of its head and face. The uterus contracted well; a slight hemorrhage only occurred, which in the debile state of the woman threw her into a fainting fit which lasted fifteen or twenty minutes. It was two months before the patient could leave her bed. Ultimately both mother and child did well. The placenta when examined was found perforated about two inches from its margin: blood was incorporated with its tissue in a space extending over two or three inches of its surface, in the centre of which the perforation existed.

"Case ii. In this case, the puncture was made in the seventh month of gestation. The 'algalie' penetrated the placenta obliquely, entering about two inches from its margin and coming out about five lines from the edge of the fœtal surface. The infant died immediately after birth, but the mother not having suffered much previous to labour, experienced none of those accidents which occurred in the instances formerly related."

On these two examples M. Gendrin would base his recommendation to puncture, which, if not delayed too long, will, he says, be successful. Where it is not, and the uterus remains inert (the inertia he attributes to delay), the practitioner may still turn the child. This method, he adds, has the advantage over the *accouchement forcé*, that it is applicable in any case, while the latter is, as we know, not always practicable before life ebbs away. We leave the further consideration of this method to our readers. That it is a valuable hint communicated in good faith and on high authority there can be little doubt.

That every aid to the practitioner in these terrible instances is a blessing, we acknowledge most thankfully. There is enough, both in the nature of the case itself, and in the source of the remedy, to make us carefully note the reasonings and facts, and to prepare us to attempt the same in similar circumstances. It is, however, untried, at least comparatively so, when opposed to the usual practice of immediate delivery by the hand. In the majority of cases, the mother is easily and instantly saved by this operation, and not unfrequently the child. Where this is practicable it should be done. The delay attending all other modes of proceeding, the dreadful anxiety of waiting for effects which we cannot calculate, the watching those points of time laden with the fiat of life or death, are persuasions to immediate action. There are occasions, however, in which the flooding is great and yet the introduction of the hand impossible; and it is in these in which we think M. Gendrin's plan should be tried. We call it M. Gendrin's plan, because it is fairly and substantively put out as a determinate course of action in certain cases; still, neither the suggestion nor the practice is his. Mr. Ingleby of Birmingham has given us a summary of this practice, at p. 157 of his book, from which it appears that Dr. F. Ramsbotham recommends rupturing the membranes in partial implantation of the placenta over the os, that Dr. Cusack performed this operation in similar cases six times; and Dr. Blundell, also, has resorted to and advises it. M. Gendrin is distinguished from these by a more distinct and a more extended recommendation of the operation, whatever may be the position of the placenta as to the os uteri.

We have conned this remarkable volume, leaf by leaf, from beginning to end. That it is the work of no common man is apparent in each sentence. It abounds in a patient display of materials, and in views in which empiricism is contending with philosophy. There is much of reading, much of observation; a due deference to the opinion of others and an honest confidence in his own. The subject is encumbered by method, stifling the free play of thoughts by the logical armour which was intended as a help and a defence. In spite of these faults, however, the work is highly creditable to M. Gendrin and to the modern French school, which is striving to unite the wide survey of the patient German with the restless and searching empiricism of the English.

